# An annotated checklist of the Chilopoda and Diplopoda (Myriapoda) of the Abrau Peninsula, northwestern Caucasus, Russia

**DOI:** 10.3897/BDJ.4.e7308

**Published:** 2016-05-19

**Authors:** Daniil I. Korobushkin, Irina I. Semenyuk, Ivan H. Tuf

**Affiliations:** ‡ A.N. Severtsov Institute of Ecology and Evolution, Russian Academy of Sciences, 33 Leninsky prospekt, Moscow, Russia; § Department of Ecology and Environmental Sciences, Faculty of Science, Palacky University, 27 Slechtitelu, Olomouc, Czech Republic

**Keywords:** Myriapoda, taxonomy, Utrish Nature Reserve, soil fauna

## Abstract

**Background:**

The Abrau Peninsula is located in northwestern Caucasus between the cities of Novorossiysk and Anapa, Krasnodar Province, Russia. This paper contains an annotated checklist of the Chilopoda and Diplopoda inhabiting the Abrau Peninsula.

**New information:**

The fauna of the Abrau Peninsula comprises 17 centipede (4 orders) and 16 millipede (6 orders) species. *Henia taurica*, hitherto known only from the Crimea, has now been reported from several localities in the studied region. The study also reveals two possibly new millipede species. Statistical analyses showed that habitat preferences of myriapod species within the Abrau Peninsula are caused by species geographic distribution pattern and microbiotope preferences.

## Introduction

The myriapod fauna of some parts of Russia still remains poorly known. This holds particularly true for the Caucasus, where most large-scale investigations of the group were performed a long time ago (Muralewitsch 1907, Muralewitsch 1926, Lohmander 1936, Zalesskaja 1978, Golovatch 1985). Most of the recent studies on myriapods in the region have been either local or focused on specific taxonomic groups (Golovatch et al. 2015). This, along with the presence of several nearly “blank spots” in this territory, emphasizes the need for further faunistic investigations in the region. One of the least explored areas in the Caucasus is the Abrau Peninsula, northwestern Caucasus and its environs, where the myriapod fauna has never been fully investigated. A particular significance of this area from a faunistic point of view is determined by the presence of a relict xerophytic sub-Mediterranean vegetation. This is the only place in Russia that harbours a Mediterranean vegetation (Seregin and Suslova 2007).

The Abrau Peninsula is located between the cities of Novorossiysk and Anapa, Krasnodar Region. Its 9065 ha area is occupied by the Utrish State Nature Reserve. The climate of the Abrau Peninsula is sub-Mediterranean with cool rainy winters without a stable snow cover and with hot dry summers. The mean annual precipitation is 500 mm, the mean July and February temperatures are 23.7°C and 2.7°C, respectively (Afonin et al. 2009). The area vegetation consists of three major belts (Seregin and Suslova 2007): (1) coastal slopes with sub-Mediterranean xerophytic forests and shrublands with pistachio (*Pistacia mutica*), juniper (*Juniperus excelsa*, *J. oxycedrus*, *J. foetidissima*), oak (*Quercus pubescens*) and oriental hornbeam (*Carpinus orientalis*); (2) piedmont and low-mountain area with a combination of mesophitic and xerophitic forests and a predominance of two oak species (*Q. pubescens*, *Q. petraea*), oriental hornbeam and junipers; (3) low mountains with mesophitic deciduous forests with a domination of oak (*Q. petraea*), hornbeam (*Carpinus caucasica*), lime (*Tilia begoniifolia*), maple (*Acer laetum*), ash (*Fraxinus excelsior*) and beech (*Fagus orientalis*). Brown-pebble forest soils dominate throughout the study area.

The abundance of millipedes and centipedes at the Abrau Peninsula averages from 784 to150 ind./m^2^, or ca. 65% and 10% of all soil macroinvertebrates (Gongalsky et al. 2004, Gongalsky et al. 2008​). Despite the existing data on the abundance and distribution of soil macroinvertebrates in the Abrau Peninsula, to the best of our knowledge there has been only one specialized publication on the myriapod fauna of Utrish Nature Reserve (Short 2015), which provides information about the geographic distribution of the genus *Lophoproctus*, including *Lophoproctus coecus* collected by Ivan H. Tuf and Daria Kuznetsova from Utrish Nature Reserve in 2013. During our field studies several presumably new species have been collected.

The goal of this paper is to summarize and expand the current knowledge on the composition and the distribution of Chilopoda and Diplopoda in the Abrau Peninsula, which would help to improve the conservation policies in the Utrish Nature Reserve and facilitate further studies on these groups in the region.

## Materials and methods

The data presented in this paper are primarily based on the field studies of the myriapods collected by the authors (a description of the sampling sites is provided in Table 1, see also Fig. 1). The samples collected by I.H. Tuf are denominated in the text by IHT, those of D.I. Korobushkin by DIK, and those of I.I. Semenyuk by IIS. In addition, our colleagues K.B. Gongalsky (marked in the text as KBG), T.Yu. Lushnikova (referred to as TYL), D.M. Kuznetsova (referred to as DMK), A.A. Panchenkov (referred to as AAP) kindly provided additional material collected in 2008–2013. Furthermore, the species data from M. Short (2015) and invertebrate ecology research (Gongalsky et al. 2008, Korobushkin 2014, Korobushkin et al. 2014) is included too. The taxonomy follows Zalesskaja (1978), Zalesskaja and Schileiko (1991), Dányi (2007), Bonato et al. (2008), Zapparoli (2009), Bonato et al. (2011), Dányi and Tuf (2012), Bonato and Minelli (2014) for Chilopoda and the classification of Shear (2011) for Diplopoda.

**Sampling**. The method of myriapod sampling differed from site to site, since the samples were collected by several authors in different years. In general sampling followed on of the four basic procedures described below.

- "Hand" – animals were collected by hand on sampling sites with the average size of 400 m^2^ by way of sorting the leaf litter, upper soil layers and woody debris.
- "Sample" – minimum 4 replicates of soil samples (25x25 cm square and 15 cm deep) were collected, then sorted by hand and sifted.
- "Corer" – 25 soil cores of 20 cm in diameter, and a depth of 15 cm were collected with the cylindrical soil corer. Samples were delivered to the laboratory in cool boxes and processed within 2-3 days, or sorted by hand immediately.
- "Whipping" – whipping the branches of trees and shrubs.

The short quoted procedure names were used in the list below to specify the method of material collection for each species.

All specimens were preserved in 70% ethanol and stored in the collection of the Zoological Museum of Lomonosov Moscow State University or in personal collections of I.I. Semenyuk, I.H. Tuf and D.I. Korobushkin.

**Figure 2.**
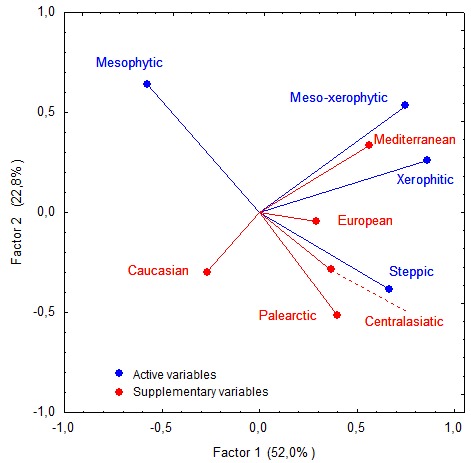
Relationship between presence of species belonging to different geographic distribution patterns (supplementary variables) in various investigated habitats (active variables) determined using the PCA.

**Data analysis**. To evaluate similarity of myriapod taxonomic composition in different microbiotopes with respect to the species' geographical distribution pattern, we applied Single Linkage Clustering analysis (Jaccard similarity index) with the presence/absence data standardization (Everitt 2011). Further, correlation between species richness of myriapods belonging to a particular geographical distribution pattern and their presence various habitats in the Abrau Peninsula was visualized using principal component analysis (PCA) (Jolliffe 2002). In this analysis geographical distribution patterns were selected the active parameters, while habitats were used as passive ones. To reduce the number of variables in the PCA analysis, in some cases myriapod species with relatively narrow distribution (e.g. East Meditteranean) were allocated in the distribution pattern covering a larger region (e.g. Mediterranean). For more details on such inclusions and generalizations please refer to figure 2. All analyses were performed using Statistica 7.0 software package.

## Checklists

### An annotated checklist of Chilopoda and Diplopoda species in the Abrau Peninsula

#### Chilopoda

#### Scutigeromorpha

#### Scutigeridae

#### Scutigera coleoptrata

(Linnaeus, 1758)

##### Materials

- **Type status:** Other material. **Occurrence:** recordedBy: AAP; Sampling: hand; sample; individualCount: 2; **Location:** country: Russia; stateProvince: Krasnodar; locality: {14}; verbatimCoordinates: 44°42'21'' N, 37°28'15'' E; 16; **Event:** eventDate: 06/2009
- **Type status:** Other material. **Occurrence:** recordedBy: DIK; Sampling: hand; individualCount: 10; **Location:** country: Russia; stateProvince: Krasnodar; locality: {14}; verbatimCoordinates: 44°42'21'' N, 37°28'15'' E; 16; **Event:** eventDate: 06-15-14
- **Type status:** Other material. **Occurrence:** recordedBy: DIK; Sampling: hand; individualCount: 2; **Location:** country: Russia; stateProvince: Krasnodar; locality: {10}; verbatimCoordinates: 44°43'31''N, 37°29'04'' E; 85; **Event:** eventDate: 06-20-12
- **Type status:** Other material. **Occurrence:** recordedBy: IHT; Sampling: hand; individualCount: 1; **Location:** country: Russia; stateProvince: Krasnodar; locality: {14}; verbatimCoordinates: 44°42'21'' N, 37°28'15'' E; 16; **Event:** eventDate: 06/2013
- **Type status:** Other material. **Occurrence:** recordedBy: IHT; Sampling: hand; individualCount: 1; **Location:** country: Russia; stateProvince: Krasnodar; locality: *P. mutica- Juniperus* shrubland; verbatimCoordinates: 44°42'2'' N, 37°28'15'' E; 16; **Event:** eventDate: 06-16-13
- **Type status:** Other material. **Occurrence:** recordedBy: IHT; Sampling: hand; individualCount: 1; **Location:** country: Russia; stateProvince: Krasnodar; locality: *P. mutica- Juniperus* shrubland on the dry slope; verbatimCoordinates: 44°42'48'' N, 37°27'58'' E; 126; **Event:** eventDate: 06-18-13
- **Type status:** Other material. **Occurrence:** recordedBy: IHT; Sampling: hand; individualCount: 2; **Location:** country: Russia; stateProvince: Krasnodar; locality: Abrau city; **Event:** eventDate: 06-20-13
- **Type status:** Other material. **Occurrence:** recordedBy: KBG, DIK, DMK, AAP, IHT; Sampling: hand, sample; individualCount: 3; **Location:** country: Russia; stateProvince: Krasnodar; locality: {3}; verbatimCoordinates: 44°42'51'' N, 37°28'45'' E; 47; **Event:** eventDate: 06/2013

##### Notes

*S. coleoptrata* is an indigenous species in the Mediterranean region, which is largely introduced by human activities throughout Europe, Asia, North America and South America (Andersson et al. 2005). In the Abrau Peninsula, the species occurs in the mesophitic and xerophitic forests and shrublands. It is also frequently recorded in urban sites.

#### Lithobiomorpha

#### Lithobiidae

#### Harpolithobius spinipes

Folkmanova, 1958

##### Materials

- **Type status:** Other material. **Occurrence:** recordedBy: KBG, DIK, AAP, IHT; Sampling: hand, sample; individualCount: 1; **Location:** country: Russia; stateProvince: Krasnodar; locality: {1}; verbatimCoordinates: 44°45'15'' N, 37°29'53'' E; 195; **Event:** eventDate: 06/2013
- **Type status:** Other material. **Occurrence:** recordedBy: KBG, DIK, DMK, AAP, IHT; Sampling: hand, sample; individualCount: 1; **Location:** country: Russia; stateProvince: Krasnodar; locality: {2}; verbatimCoordinates: 44°44'13'' N, 37°28'46'' E; 153; **Event:** eventDate: 06/2013
- **Type status:** Other material. **Occurrence:** recordedBy: KBG, DIK, DMK, AAP, IHT; Sampling: hand, sample; individualCount: 1; **Location:** country: Russia; stateProvince: Krasnodar; locality: {2}; verbatimCoordinates: 44°44'13'' N, 37°28'46'' E; 153; **Event:** eventDate: 06/2013

##### Notes

This species is widespread in the Caucasus (Zalesskaja 1978). In the Abrau Peninsula, the specimens were collected from the leaflitter layer.

#### Lithobius (Monotarsobius) curtipes

C.L. Koch, 1847

##### Materials

- **Type status:** Other material. **Occurrence:** recordedBy: KBG, DIK; Sampling: sample; individualCount: 2; **Location:** country: Russia; stateProvince: Krasnodar; locality: {5}; verbatimCoordinates: 44°42'38'' N, 37°27'31'' E; 10; **Event:** eventDate: 06/2013
- **Type status:** Other material. **Occurrence:** recordedBy: KBG, DIK; Sampling: sample; individualCount: 3; **Location:** country: Russia; stateProvince: Krasnodar; locality: {6}; verbatimCoordinates: 44°42'34'' N, 37°27'25'' E; 6; **Event:** eventDate: 06/2013

##### Notes

The most common and abundant species in the European part of Russia (Zalesskaja 1978). It is known from the Caucasus in the south to the Altai Mountains in the east. The species also occurs north of the Polar Circle (Zalesskaja 1978, Palmén 1988). In the Caucasus, it was found in the upper soil layer (0-10 сm) and, less frequently, in the leaflitter layer of coniferous and mixed forests. The species is also known from steppes.

#### Lithobius (Monotarsobius) ferganensis

(Trotzina, 1894)

##### Materials

- **Type status:** Other material. **Occurrence:** recordedBy: DMK; Sampling: hand; individualCount: 3; **Location:** country: Russia; stateProvince: Krasnodar; locality: {10}; verbatimCoordinates: 44°43'31''N, 37°29'04'' E; 85; **Event:** eventDate: 06-20-12
- **Type status:** Other material. **Occurrence:** recordedBy: IHT; Sampling: hand; individualCount: 1; **Location:** country: Russia; stateProvince: Krasnodar; locality: {14}; verbatimCoordinates: 44°42'21'' N, 37°28'15'' E; 16; **Event:** eventDate: 06/2013
- **Type status:** Other material. **Occurrence:** recordedBy: IHT; Sampling: hand; individualCount: 6; **Location:** country: Russia; stateProvince: Krasnodar; locality: {2}; verbatimCoordinates: 44°44'13'' N, 37°28'46'' E; 153; **Event:** eventDate: 06/2013
- **Type status:** Other material. **Occurrence:** recordedBy: IHT; Sampling: hand; individualCount: 4; **Location:** country: Russia; stateProvince: Krasnodar; locality: {13}; verbatimCoordinates: 44°42'39''N, 37°28'37'' E; 31; **Event:** eventDate: 06-18-13
- **Type status:** Other material. **Occurrence:** recordedBy: IHT; Sampling: hand; individualCount: 2; **Location:** country: Russia; stateProvince: Krasnodar; locality: *P. mutica - Juniperus* shrubland on the dry slope; verbatimCoordinates: 44°42'48'' N, 37°27'58'' E; 126; **Event:** eventDate: 06-18-13
- **Type status:** Other material. **Occurrence:** recordedBy: IHT; Sampling: hand; individualCount: 5; **Location:** country: Russia; stateProvince: Krasnodar; locality: {8}; verbatimCoordinates: 44°45'14'' N, 37°27'26'' E; 308; **Event:** eventDate: 06-19-13
- **Type status:** Other material. **Occurrence:** recordedBy: IHT; Sampling: hand; individualCount: 1; **Location:** country: Russia; stateProvince: Krasnodar; locality: Abrau city; **Event:** eventDate: 06-20-13
- **Type status:** Other material. **Occurrence:** recordedBy: KBG, DIK, AAP, IHT; Sampling: hand, sample; individualCount: 5; **Location:** country: Russia; stateProvince: Krasnodar; locality: {1}; verbatimCoordinates: 44°45'15'' N, 37°29'53'' E; 195; **Event:** eventDate: 06/2013
- **Type status:** Other material. **Occurrence:** recordedBy: KBG, DIK, DMK, AAP, IHT; Sampling: hand, sample; individualCount: 6; **Location:** country: Russia; stateProvince: Krasnodar; locality: {2}; verbatimCoordinates: 44°44'13'' N, 37°28'46'' E; 153; **Event:** eventDate: 06/2013
- **Type status:** Other material. **Occurrence:** recordedBy: KBG, DIK, DMK, AAP, IHT; Sampling: hand, sample; individualCount: 4; **Location:** country: Russia; stateProvince: Krasnodar; locality: {3}; verbatimCoordinates: 44°42'51'' N, 37°28'45'' E; 47; **Event:** eventDate: 06/2013
- **Type status:** Other material. **Occurrence:** recordedBy: KBG, DIK, DMK, AAP, IHT; Sampling: hand, sample; individualCount: 6; **Location:** country: Russia; stateProvince: Krasnodar; locality: {3}; verbatimCoordinates: 44°42'51'' N, 37°28'45'' E; 47; **Event:** eventDate: 06/2013
- **Type status:** Other material. **Occurrence:** recordedBy: KBG, DIK, DMK, AAP, IHT; Sampling: hand, sample; individualCount: 9; **Location:** country: Russia; stateProvince: Krasnodar; locality: {11}; verbatimCoordinates: 44°42'56''N, 37°28'50'' E; 54; **Event:** eventDate: 06-15-13
- **Type status:** Other material. **Occurrence:** recordedBy: KBG, DIK, DMK, AAP, IHT; Sampling: hand, sample; individualCount: 4; **Location:** country: Russia; stateProvince: Krasnodar; locality: {12}; verbatimCoordinates: 44°42'48''N, 37°28'39'' E; 50; **Event:** eventDate: 06-16-13
- **Type status:** Other material. **Occurrence:** recordedBy: KBG, DIK, DMK, AAP, IHT; Sampling: hand, sample; individualCount: 7; **Location:** country: Russia; stateProvince: Krasnodar; locality: {13}; verbatimCoordinates: 44°42'39''N, 37°28'37'' E; 31; **Event:** eventDate: 06-17-13
- **Type status:** Other material. **Occurrence:** recordedBy: TYL; Sampling: Corer; individualCount: 6; **Location:** country: Russia; stateProvince: Krasnodar; locality: {10}; verbatimCoordinates: 44°43'31''N, 37°29'04'' E; 85; **Event:** eventDate: 06/2010
- **Type status:** Other material. **Occurrence:** recordedBy: TYL; Sampling: Corer; individualCount: 6; **Location:** country: Russia; stateProvince: Krasnodar; locality: *A. laetum - F. excelsior* forest with *Q. petraea*; **Event:** eventDate: 06/2010
- **Type status:** Other material. **Occurrence:** recordedBy: TYL; Sampling: Corer; individualCount: 1; **Location:** country: Russia; stateProvince: Krasnodar; locality: *C. caucasica* forest with *F. excelsior, Q. petraea* and *T. begoniifolia*; **Event:** eventDate: 06/2010
- **Type status:** Other material. **Occurrence:** recordedBy: TYL; Sampling: Corer; individualCount: 2; **Location:** country: Russia; stateProvince: Krasnodar; locality: *Q. pubescens* - *C. orientalis* forest; **Event:** eventDate: 06/2010
- **Type status:** Other material. **Occurrence:** recordedBy: TYL; Sampling: Corer; individualCount: 2; **Location:** country: Russia; stateProvince: Krasnodar; locality: *T. begoniifolia - Q. petraea* forest; **Event:** eventDate: 06/2010
- **Type status:** Other material. **Occurrence:** recordedBy: TYL; Sampling: Corer; individualCount: 1; **Location:** country: Russia; stateProvince: Krasnodar; locality: Tangle of *Paliurus spina-christi*; **Event:** eventDate: 06/2010
- **Type status:** Other material. **Occurrence:** recordedBy: TYL; Sampling: Corer; individualCount: 1; **Location:** country: Russia; stateProvince: Krasnodar; locality: *C. caucasica-F. orientalis* forest; verbatimCoordinates: 44°43'41'' N, 37°29'25'' E; 188; **Event:** eventDate: 06-11-10
- **Type status:** Other material. **Occurrence:** recordedBy: TYL; Sampling: Corer; individualCount: 1; **Location:** country: Russia; stateProvince: Krasnodar; locality: *C. caucasica-F. orientalis* forest; **Event:** eventDate: 06-11-10
- **Type status:** Other material. **Occurrence:** recordedBy: TYL; Sampling: Corer; individualCount: 6; **Location:** country: Russia; stateProvince: Krasnodar; locality: *C. orientalis - Q. pubescens* forest with *T. begoniifolia* and *F. excelsior*; **Event:** eventDate: 06-13-10
- **Type status:** Other material. **Occurrence:** recordedBy: TYL; Sampling: Corer; individualCount: 2; **Location:** country: Russia; stateProvince: Krasnodar; locality: *Q. pubescens* - *Pinus pityusa* forest; **Event:** eventDate: 06-13-10
- **Type status:** Other material. **Occurrence:** recordedBy: TYL; Sampling: Corer; individualCount: 4; **Location:** country: Russia; stateProvince: Krasnodar; locality: Open area north of the lake Sukhoy Liman; **Event:** eventDate: 06-14-10
- **Type status:** Other material. **Occurrence:** recordedBy: TYL; Sampling: Corer; individualCount: 1; **Location:** country: Russia; stateProvince: Krasnodar; locality: *Q. petraea, F. orientalis* forest with *T. begoniifolia*; **Event:** eventDate: 06-16-10
- **Type status:** Other material. **Occurrence:** recordedBy: TYL; Sampling: Corer; individualCount: 5; **Location:** country: Russia; stateProvince: Krasnodar; locality: *Q. petraea - F. excelsior* forest; **Event:** eventDate: 06-17-10

##### Notes

This species is widespread in Central Asia, the Caucasus and the Crimea reaching the Chinese Karakoram in the east, and Greece and Romania in the west (Eason 1997, Stoev 2004). It is often found at high elevations, and shows preference for meadows, sometimes also recorded from caves (Zalesskaja 1978). In the Abrau Peninsula, *L. ferganensis* occurs in a wide range of habitats: from mountanious deciduous forests (e.g. *Q. petraea, F. orientalis, C. caucasica forest*) to *P. mutica - Juniperus* shrubland on dry slopes and open areas. The specimens were collected mainly from the upper soil and compressed FH layers, as well as under the bark of decaying trees.

#### Lithobius forficatus

(Linnaeus, 1758)

##### Materials

- **Type status:** Other material. **Occurrence:** recordedBy: DMK; Sampling: hand; individualCount: 1; **Location:** country: Russia; stateProvince: Krasnodar; locality: {10}; verbatimCoordinates: 44°43'31''N, 37°29'04'' E; 85; **Event:** eventDate: 06-20-13
- **Type status:** Other material. **Occurrence:** recordedBy: KBG, DIK; Sampling: sample; individualCount: 3; **Location:** country: Russia; stateProvince: Krasnodar; locality: {2}; verbatimCoordinates: 44°44'13'' N, 37°28'46'' E; 153; **Event:** eventDate: 06/2013
- **Type status:** Other material. **Occurrence:** recordedBy: KBG, DIK, DMK, AAP, IHT; Sampling: hand, sample; individualCount: 3; **Location:** country: Russia; stateProvince: Krasnodar; locality: {1}; verbatimCoordinates: 44°45'15'' N, 37°29'53'' E; 195; **Event:** eventDate: 06/2013

##### Notes

*L. forficatus* is an eurytopic species, showing a pan-Holarctic distribution pattern, widely distributed from Great Britain to Turkey and Georgia, the eastern boundary of its range reaching the Ural Mountains (Zalesskaja 1978). The species has also been introduced in the Far East, Africa, North and South America, etc. (Zapparoli 2009). In the studied area, the species occurs in the leaf litter layer, under the stones and under the bark of dead trees in deciduous forests.

#### Lithobius mutabilis

L. Koch, 1862

##### Materials

- **Type status:** Other material. **Occurrence:** recordedBy: DMK; Sampling: hand; individualCount: 7; **Location:** country: Russia; stateProvince: Krasnodar; locality: {10}; verbatimCoordinates: 44°43'31''N, 37°29'04'' E; 85; **Event:** eventDate: 06-20-12
- **Type status:** Other material. **Occurrence:** recordedBy: DMK; Sampling: hand; individualCount: 2; **Location:** country: Russia; stateProvince: Krasnodar; locality: {10}; verbatimCoordinates: 44°43'31''N, 37°29'04'' E; 85; **Event:** eventDate: 06-14-13
- **Type status:** Other material. **Occurrence:** recordedBy: IHT; Sampling: hand; individualCount: 2; **Location:** country: Russia; stateProvince: Krasnodar; locality: {14}; verbatimCoordinates: 44°42'21'' N, 37°28'15'' E; 16; **Event:** eventDate: 06/2013
- **Type status:** Other material. **Occurrence:** recordedBy: IHT; Sampling: hand; individualCount: 2; **Location:** country: Russia; stateProvince: Krasnodar; locality: {2}; verbatimCoordinates: 44°44'13'' N, 37°28'46'' E; 153; **Event:** eventDate: 06/2013
- **Type status:** Other material. **Occurrence:** recordedBy: IHT; Sampling: hand; individualCount: 3; **Location:** country: Russia; stateProvince: Krasnodar; locality: {8}; verbatimCoordinates: 44°45'14'' N, 37°27'26'' E; 308; **Event:** eventDate: 06-19-13
- **Type status:** Other material. **Occurrence:** recordedBy: KBG, DIK; Sampling: sample; individualCount: 1; **Location:** country: Russia; stateProvince: Krasnodar; locality: open areas north of the lake Sukhoy Liman; **Event:** eventDate: 06-14-10
- **Type status:** Other material. **Occurrence:** recordedBy: KBG, DIK, AAP, IHT; Sampling: hand, sample; individualCount: 1; **Location:** country: Russia; stateProvince: Krasnodar; locality: {1}; verbatimCoordinates: 44°45'15'' N, 37°29'53'' E; 195; **Event:** eventDate: 06/2013
- **Type status:** Other material. **Occurrence:** recordedBy: KBG, DIK, DMK, AAP, IHT; Sampling: hand, sample; individualCount: 2; **Location:** country: Russia; stateProvince: Krasnodar; locality: *C. caucasica - F. orientalis* forest; verbatimCoordinates: 44°43'41'' N, 37°29'25'' E; 188; **Event:** eventDate: 06-11-10
- **Type status:** Other material. **Occurrence:** recordedBy: KBG, DIK, DMK, AAP, IHT; Sampling: hand, sample; individualCount: 4; **Location:** country: Russia; stateProvince: Krasnodar; locality: {2}; verbatimCoordinates: 44°44'13'' N, 37°28'46'' E; 153; **Event:** eventDate: 06/2013
- **Type status:** Other material. **Occurrence:** recordedBy: KBG, DIK, DMK, AAP, IHT; Sampling: hand, sample; individualCount: 1; **Location:** country: Russia; stateProvince: Krasnodar; locality: {3}; verbatimCoordinates: 44°42'51'' N, 37°28'45'' E; 47; **Event:** eventDate: 06/2013
- **Type status:** Other material. **Occurrence:** recordedBy: KBG, DIK, DMK, AAP, IHT; Sampling: hand, sample; individualCount: 5; **Location:** country: Russia; stateProvince: Krasnodar; locality: {3}; verbatimCoordinates: 44°42'51'' N, 37°28'45'' E; 47; **Event:** eventDate: 06/2013
- **Type status:** Other material. **Occurrence:** recordedBy: KBG, DIK, DMK, AAP, IHT; Sampling: hand, sample; individualCount: 10; **Location:** country: Russia; stateProvince: Krasnodar; locality: {11}; verbatimCoordinates: 44°42'56''N, 37°28'50'' E; 54; **Event:** eventDate: 06-15-13
- **Type status:** Other material. **Occurrence:** recordedBy: KBG, DIK, DMK, AAP, IHT; Sampling: hand, sample; individualCount: 10; **Location:** country: Russia; stateProvince: Krasnodar; locality: {11}; verbatimCoordinates: 44°42'56''N, 37°28'50'' E; 54; **Event:** eventDate: 06-15-13
- **Type status:** Other material. **Occurrence:** recordedBy: KBG, DIK, DMK, AAP, IHT; Sampling: hand, sample; individualCount: 2; **Location:** country: Russia; stateProvince: Krasnodar; locality: {12}; verbatimCoordinates: 44°42'48''N, 37°28'39'' E; 50; **Event:** eventDate: 06-16-13
- **Type status:** Other material. **Occurrence:** recordedBy: KBG, DIK, DMK, AAP, IHT; Sampling: hand, sample; individualCount: 6; **Location:** country: Russia; stateProvince: Krasnodar; locality: {13}; verbatimCoordinates: 44°42'39''N, 37°28'37'' E; 31; **Event:** eventDate: 06-17-13
- **Type status:** Other material. **Occurrence:** recordedBy: KBG, DIK, DMK, AAP, IHT; Sampling: hand, sample; individualCount: 11; **Location:** country: Russia; stateProvince: Krasnodar; locality: {13}; verbatimCoordinates: 44°42'39''N, 37°28'37'' E; 31; **Event:** eventDate: 06-18-13
- **Type status:** Other material. **Occurrence:** recordedBy: TYL; Sampling: Corer; individualCount: 1; **Location:** country: Russia; stateProvince: Krasnodar; locality: {10}; verbatimCoordinates: 44°43'31''N, 37°29'04'' E; 85; **Event:** eventDate: 06/2011
- **Type status:** Other material. **Occurrence:** recordedBy: TYL; Sampling: Corer; individualCount: 2; **Location:** country: Russia; stateProvince: Krasnodar; locality: *A. laetum - F. excelsior* forest with *Q. petraea*; **Event:** eventDate: 06/2011
- **Type status:** Other material. **Occurrence:** recordedBy: TYL; Sampling: Corer; individualCount: 2; **Location:** country: Russia; stateProvince: Krasnodar; locality: *T. begoniifolia - Q. petraea* forest; **Event:** eventDate: 06/2011

##### Notes

This species is known from mesophilous woodlands (e.g. beech and fir-beech forests) in Central Europe, Italy, Bulgaria, Greece, the southern part of Russia and Ukraine, including the Crimea (Zalesskaja 1978, Stoev 2004, Zapparoli 2009). In the Abrau Peninsula the species occurs in different habitats, from open areas to deciduous forests, where it inhabits top soil (0-20 cm) and leaf litter layers.

#### Lithobius peregrinus

Latzel, 1880

##### Materials

- **Type status:** Other material. **Occurrence:** recordedBy: DIK, AAP, IHT; Sampling: hand; sample; individualCount: 5; **Location:** country: Russia; stateProvince: Krasnodar; locality: {4}; verbatimCoordinates: 44°42'50'' N, 37°27'01'' E; 8; **Event:** eventDate: 06/2013
- **Type status:** Other material. **Occurrence:** recordedBy: DMK; Sampling: hand; individualCount: 8; **Location:** country: Russia; stateProvince: Krasnodar; locality: {10}; verbatimCoordinates: 44°43'31''N, 37°29'04'' E; 85; **Event:** eventDate: 06-20-13
- **Type status:** Other material. **Occurrence:** recordedBy: IHT; Sampling: hand; individualCount: 2; **Location:** country: Russia; stateProvince: Krasnodar; locality: {13}; verbatimCoordinates: 44°42'39''N, 37°28'37'' E; 31; **Event:** eventDate: 06/2013
- **Type status:** Other material. **Occurrence:** recordedBy: IHT; Sampling: hand; individualCount: 3; **Location:** country: Russia; stateProvince: Krasnodar; locality: {14}; verbatimCoordinates: 44°42'21'' N, 37°28'15'' E; 16; **Event:** eventDate: 06/2013
- **Type status:** Other material. **Occurrence:** recordedBy: IHT; Sampling: hand; individualCount: 4; **Location:** country: Russia; stateProvince: Krasnodar; locality: {2}; verbatimCoordinates: 44°44'13'' N, 37°28'46'' E; 153; **Event:** eventDate: 06/2013
- **Type status:** Other material. **Occurrence:** recordedBy: IHT; Sampling: hand; individualCount: 2; **Location:** country: Russia; stateProvince: Krasnodar; locality: *P. mutica - Juniperus* shrubland on the dry slope; verbatimCoordinates: 44°42'48'' N, 37°27'58'' E; 126; **Event:** eventDate: 06-18-13
- **Type status:** Other material. **Occurrence:** recordedBy: IHT; Sampling: hand; individualCount: 4; **Location:** country: Russia; stateProvince: Krasnodar; locality: {8}; verbatimCoordinates: 44°45'14'' N, 37°27'26'' E; 308; **Event:** eventDate: 06-19-13
- **Type status:** Other material. **Occurrence:** recordedBy: KBG, DIK; Sampling: sample; individualCount: 6; **Location:** country: Russia; stateProvince: Krasnodar; locality: {5}; verbatimCoordinates: 44°42'38'' N, 37°27'31'' E; 10; **Event:** eventDate: 06/2009
- **Type status:** Other material. **Occurrence:** recordedBy: KBG, DIK; Sampling: sample; individualCount: 7; **Location:** country: Russia; stateProvince: Krasnodar; locality: {8}; verbatimCoordinates: 44°45'14'' N, 37°27'26'' E; 308; **Event:** eventDate: 06/2009
- **Type status:** Other material. **Occurrence:** recordedBy: KBG, DIK, DMK, AAP, IHT; Sampling: hand, sample; individualCount: 5; **Location:** country: Russia; stateProvince: Krasnodar; locality: {2}; verbatimCoordinates: 44°44'13'' N, 37°28'46'' E; 153; **Event:** eventDate: 06/2013
- **Type status:** Other material. **Occurrence:** recordedBy: KBG, DIK, DMK, AAP, IHT; Sampling: hand, sample; individualCount: 1; **Location:** country: Russia; stateProvince: Krasnodar; locality: {3}; verbatimCoordinates: 44°42'51'' N, 37°28'45'' E; 47; **Event:** eventDate: 06/2013
- **Type status:** Other material. **Occurrence:** recordedBy: KBG, DIK, DMK, AAP, IHT; Sampling: hand, sample; individualCount: 3; **Location:** country: Russia; stateProvince: Krasnodar; locality: {13}; verbatimCoordinates: 44°42'39''N, 37°28'37'' E; 31; **Event:** eventDate: 06-07-13
- **Type status:** Other material. **Occurrence:** recordedBy: KBG, DIK, DMK, AAP, IHT; Sampling: hand, sample; individualCount: 2; **Location:** country: Russia; stateProvince: Krasnodar; locality: {11}; verbatimCoordinates: 44°42'56''N, 37°28'50'' E; 54; **Event:** eventDate: 06-15-13
- **Type status:** Other material. **Occurrence:** recordedBy: TYL; Sampling: Corer; individualCount: 1; **Location:** country: Russia; stateProvince: Krasnodar; locality: *C. caucasica - F. orientalis* forest; **Event:** eventDate: 06-11-10

##### Notes

*L. peregrinus* displays a mostly Mediterranean distribution pattern. The species is known from Italy, Croatia, Montenegro, Serbia, Macedonia, Albania, Greece, Bulgaria, Russian and Georgian sectors of Caucasus. It is also introduced into Great Britain, Panama, Bermuda Islands, and South Africa (Zalesskaja 1978, Zapparoli 1992). In the Abrau Peninsula it inhabits litter and microbiotopes under the bark of dead trees of deciduous forests.

#### Scolopendromorpha

#### Cryptopidae

#### Cryptops anomalans

Newport, 1844

##### Materials

- **Type status:** Other material. **Occurrence:** recordedBy: DMK; Sampling: hand; individualCount: 2; **Location:** country: Russia; stateProvince: Krasnodar; locality: {10}; verbatimCoordinates: 44°43'31''N, 37°29'04'' E; 85; **Event:** eventDate: 06-14-13
- **Type status:** Other material. **Occurrence:** recordedBy: DMK; Sampling: hand; individualCount: 3; **Location:** country: Russia; stateProvince: Krasnodar; locality: {10}; verbatimCoordinates: 44°43'31''N, 37°29'04'' E; 85; **Event:** eventDate: 06-14-13
- **Type status:** Other material. **Occurrence:** recordedBy: IHT; Sampling: hand; individualCount: 5; **Location:** country: Russia; stateProvince: Krasnodar; locality: {1}; verbatimCoordinates: 44°45'15'' N, 37°29'53'' E; 195; **Event:** eventDate: 06/2013
- **Type status:** Other material. **Occurrence:** recordedBy: IHT; Sampling: hand; individualCount: 2; **Location:** country: Russia; stateProvince: Krasnodar; locality: {13}; verbatimCoordinates: 44°42'39''N, 37°28'37'' E; 31; **Event:** eventDate: 06/2013
- **Type status:** Other material. **Occurrence:** recordedBy: IHT; Sampling: hand; individualCount: 5; **Location:** country: Russia; stateProvince: Krasnodar; locality: {2}; verbatimCoordinates: 44°44'13'' N, 37°28'46'' E; 153; **Event:** eventDate: 06/2013
- **Type status:** Other material. **Occurrence:** recordedBy: IHT; Sampling: hand; individualCount: 6; **Location:** country: Russia; stateProvince: Krasnodar; locality: {8}; verbatimCoordinates: 44°45'14'' N, 37°27'26'' E; 308; **Event:** eventDate: 06/2013
- **Type status:** Other material. **Occurrence:** recordedBy: IHT; Sampling: hand; individualCount: 2; **Location:** country: Russia; stateProvince: Krasnodar; locality: P. mutica - Juniperus shrubland on the dry slope; verbatimCoordinates: 44°42'48'' N, 37°27'58'' E; 126; **Event:** eventDate: 06-18-13
- **Type status:** Other material. **Occurrence:** recordedBy: KBG, DIK, DMK, AAP, IHT; Sampling: hand, sample; individualCount: 4; **Location:** country: Russia; stateProvince: Krasnodar; locality: {3}; verbatimCoordinates: 44°42'51'' N, 37°28'45'' E; 47; **Event:** eventDate: 06/2013
- **Type status:** Other material. **Occurrence:** recordedBy: KBG, DIK, DMK, AAP, IHT; Sampling: hand, sample; individualCount: 1; **Location:** country: Russia; stateProvince: Krasnodar; locality: {3}; verbatimCoordinates: 44°42'51'' N, 37°28'45'' E; 47; **Event:** eventDate: 06/2013
- **Type status:** Other material. **Occurrence:** recordedBy: KBG, DIK, DMK, AAP, IHT; Sampling: hand, sample; individualCount: 5; **Location:** country: Russia; stateProvince: Krasnodar; locality: {11}; verbatimCoordinates: 44°42'56''N, 37°28'50'' E; 54; **Event:** eventDate: 06-15-13
- **Type status:** Other material. **Occurrence:** recordedBy: KBG, DIK, DMK, AAP, IHT; Sampling: hand, sample; individualCount: 3; **Location:** country: Russia; stateProvince: Krasnodar; locality: {12}; verbatimCoordinates: 44°42'48''N, 37°28'39'' E; 50; **Event:** eventDate: 06-16-13
- **Type status:** Other material. **Occurrence:** recordedBy: KBG, DIK, DMK, AAP, IHT; Sampling: hand, sample; individualCount: 2; **Location:** country: Russia; stateProvince: Krasnodar; locality: {13}; verbatimCoordinates: 44°42'39''N, 37°28'37'' E; 31; **Event:** eventDate: 06-17-13
- **Type status:** Other material. **Occurrence:** recordedBy: TYL; Sampling: Corer; individualCount: 2; **Location:** country: Russia; stateProvince: Krasnodar; locality: *Q. pubescens - C. orientalis* forest; verbatimCoordinates: 44°43'41'' N, 37°29'25'' E; 188; **Event:** eventDate: 06-08-10
- **Type status:** Other material. **Occurrence:** recordedBy: TYL; Sampling: Corer; individualCount: 4; **Location:** country: Russia; stateProvince: Krasnodar; locality: *C. caucasica - F. orientalis* forest; **Event:** eventDate: 06-11-10
- **Type status:** Other material. **Occurrence:** recordedBy: TYL; Sampling: Corer; individualCount: 2; **Location:** country: Russia; stateProvince: Krasnodar; locality: *Q. petraea, F. orientalis* forest with *T. begoniifolia*; **Event:** eventDate: 06-16-10
- **Type status:** Other material. **Occurrence:** recordedBy: TYL; Sampling: Corer; individualCount: 1; **Location:** country: Russia; stateProvince: Krasnodar; locality: {10}; verbatimCoordinates: 44°43'31''N, 37°29'04'' E; 85; **Event:** eventDate: 06/2011
- **Type status:** Other material. **Occurrence:** recordedBy: TYL; Sampling: Corer; individualCount: 1; **Location:** country: Russia; stateProvince: Krasnodar; locality: *A. laetum - F. excelsior* forest with *Q. petraea*; **Event:** eventDate: 06/2011

##### Notes

This South European species is distributed from Spain to Turkey and southern Ukraine (Askania Nova Biosphere Reserve). It is especially common in the Crimea Peninsula (Zalesskaja and Schileiko 1991, Zapparoli and Iorio 2012). The species has also been recorded in Central Europe and has been introduced into Great Britain and, probably, North America (Zapparoli and Iorio 2012).​ In the study area it was found beneath the stones and in the soil.

#### Cryptops hortensis

(Donovan, 1810)

##### Materials

- **Type status:** Other material. **Occurrence:** recordedBy: KBG, DIK, AAP, IHT; Sampling: hand, sample; individualCount: 18; **Location:** country: Russia; stateProvince: Krasnodar; locality: {1}; verbatimCoordinates: 44°45'15'' N, 37°29'53'' E; 195; **Event:** eventDate: 06/2013
- **Type status:** Other material. **Occurrence:** recordedBy: KBG, DIK, DMK, AAP, IHT; Sampling: hand, sample; individualCount: 18; **Location:** country: Russia; stateProvince: Krasnodar; locality: {2}; verbatimCoordinates: 44°44'13'' N, 37°28'46'' E; 153; **Event:** eventDate: 06/2013

##### Notes

This Centralasiatic-European species ranges from Great Britain and Iceland in the north to Morocco and Turkey in the south and Uzbekistan and Tajikistan in the East. The species has also been introduced into North America, some Atlantic and Pacific islands (Zapparoli 2009), and is widespread in the Caucasus (Zalesskaja and Schileiko 1991). In the Abrau Peninsula, the species occurs in the leaf litter and soil layer, under the bark of dead broadleaf trees (*Q. petraea, F. orientalis, C. caucasica*) forests.

#### Scolopendridae

#### Scolopendra cingulata

Latreille, 1829

##### Materials

- **Type status:** Other material. **Occurrence:** recordedBy: AAP; Sampling: hand; sample; individualCount: 2; **Location:** country: Russia; stateProvince: Krasnodar; locality: {14}; verbatimCoordinates: 44°42'21'' N, 37°28'15'' E; 16; **Event:** eventDate: 06/2011
- **Type status:** Other material. **Occurrence:** recordedBy: KBG, DIK; Sampling: sample; individualCount: 4; **Location:** country: Russia; stateProvince: Krasnodar; locality: {6}; verbatimCoordinates: 44°42'34'' N, 37°27'25'' E; 6; **Event:** eventDate: 06/2009
- **Type status:** Other material. **Occurrence:** recordedBy: KBG, DIK; Sampling: sample; individualCount: 4; **Location:** country: Russia; stateProvince: Krasnodar; locality: {7}; verbatimCoordinates: 44°42'29'', 37°27'28'' E; 2; **Event:** eventDate: 06/2009
- **Type status:** Other material. **Occurrence:** recordedBy: KBG, DIK; Sampling: sample; individualCount: 4; **Location:** country: Russia; stateProvince: Krasnodar; locality: {9}; verbatimCoordinates: 44°41'44'' N, 37°29'06'' E; 9; **Event:** eventDate: 06/2009
- **Type status:** Other material. **Occurrence:** recordedBy: KBG, DIK, DMK, AAP, IHT; Sampling: hand, sample; individualCount: 1; **Location:** country: Russia; stateProvince: Krasnodar; locality: {3}; verbatimCoordinates: 44°42'51'' N, 37°28'45'' E; 47; **Event:** eventDate: 06/2008
- **Type status:** Other material. **Occurrence:** recordedBy: TYL; Sampling: Corer; individualCount: 1; **Location:** country: Russia; stateProvince: Krasnodar; locality: Q. pubescens - C. orientalis; verbatimCoordinates: 44°43'41'' N, 37°29'25'' E; 188; **Event:** eventDate: 06-11-10
- **Type status:** Other material. **Occurrence:** recordedBy: TYL; Sampling: Corer; individualCount: 1; **Location:** country: Russia; stateProvince: Krasnodar; locality: *Juniperus - Quercus* shrubland; verbatimCoordinates: 44°43'14'' N, 37°29'11'' E; 107; **Event:** eventDate: 06-17-10

##### Notes

This species is widely distributed in the Mediterranean. It is common in Crimea and the Caucasus, known from Iran, Turkey and Middle Asia (Zalesskaja and Schileiko 1991, Zapparoli 2002). In the studied region, the species occurs almost exclusively in ecosystems with xerophytic sub-Mediterranean vegetation.

#### Geophilomorpha

#### Dignathodontidae

#### Henia (Meinertia) taurica

(Sseliwanoff, 1884)

##### Materials

- **Type status:** Other material. **Occurrence:** recordedBy: DIK, AAP, IHT; Sampling: hand; sample; individualCount: 1; **Location:** country: Russia; stateProvince: Krasnodar; locality: {4}; verbatimCoordinates: 44°42'50'' N, 37°27'01'' E; 8; **Event:** eventDate: 06/2013
- **Type status:** Other material. **Occurrence:** recordedBy: DMK; Sampling: hand; individualCount: 3; **Location:** country: Russia; stateProvince: Krasnodar; locality: {10}; verbatimCoordinates: 44°43'31''N, 37°29'04'' E; 85; **Event:** eventDate: 06-14-13
- **Type status:** Other material. **Occurrence:** recordedBy: DMK; Sampling: hand; individualCount: 2; **Location:** country: Russia; stateProvince: Krasnodar; locality: {10}; verbatimCoordinates: 44°43'31''N, 37°29'04'' E; 85; **Event:** eventDate: 06-14-13
- **Type status:** Other material. **Occurrence:** recordedBy: IHT; Sampling: hand; individualCount: 1; **Location:** country: Russia; stateProvince: Krasnodar; locality: {13}; verbatimCoordinates: 44°42'39''N, 37°28'37'' E; 31; **Event:** eventDate: 06/2013
- **Type status:** Other material. **Occurrence:** recordedBy: IHT; Sampling: hand; individualCount: 2; **Location:** country: Russia; stateProvince: Krasnodar; locality: {8}; verbatimCoordinates: 44°45'14'' N, 37°27'26'' E; 308; **Event:** eventDate: 06-19-13
- **Type status:** Other material. **Occurrence:** recordedBy: KBG, DIK, AAP, IHT; Sampling: hand, sample; individualCount: 1; **Location:** country: Russia; stateProvince: Krasnodar; locality: {1}; verbatimCoordinates: 44°45'15'' N, 37°29'53'' E; 195; **Event:** eventDate: 06/2013
- **Type status:** Other material. **Occurrence:** recordedBy: KBG, DIK, DMK, AAP, IHT; Sampling: hand, sample; individualCount: 2; **Location:** country: Russia; stateProvince: Krasnodar; locality: {2}; verbatimCoordinates: 44°44'13'' N, 37°28'46'' E; 153; **Event:** eventDate: 06/2013
- **Type status:** Other material. **Occurrence:** recordedBy: KBG, DIK, DMK, AAP, IHT; Sampling: hand, sample; individualCount: 3; **Location:** country: Russia; stateProvince: Krasnodar; locality: {13}; verbatimCoordinates: 44°42'39''N, 37°28'37'' E; 31; **Event:** eventDate: 06-17-13
- **Type status:** Other material. **Occurrence:** recordedBy: TYL; Sampling: Corer; individualCount: 3; **Location:** country: Russia; stateProvince: Krasnodar; locality: {10}; verbatimCoordinates: 44°43'31''N, 37°29'04'' E; 85; **Event:** eventDate: 06/2011
- **Type status:** Other material. **Occurrence:** recordedBy: TYL; Sampling: Corer; individualCount: 4; **Location:** country: Russia; stateProvince: Krasnodar; locality: *A. laetum - F. excelsior* forest with *Q. petraea*; **Event:** eventDate: 06/2011
- **Type status:** Other material. **Occurrence:** recordedBy: TYL; Sampling: Corer; individualCount: 2; **Location:** country: Russia; stateProvince: Krasnodar; locality: *T. begoniifolia-Q. petraea* forest; **Event:** eventDate: 06/2011
- **Type status:** Other material. **Occurrence:** recordedBy: TYL; Sampling: Corer; individualCount: 1; **Location:** country: Russia; stateProvince: Krasnodar; locality: *Q. petraea, F. orientalis* forest with *T. begoniifolia*; **Event:** eventDate: 06-16-13

##### Notes

This species was originally described by Seliwanoff in the Crimea Peninsula (Seliwanoff 1884) and, until now, has been known as the endemic to Crimea. In the study area it was found mainly beneath the stones of deciduous forests.

#### Geophilidae

#### Clinopodes caucasicus

(Sseliwanoff, 1884)

##### Materials

- **Type status:** Other material. **Occurrence:** recordedBy: DMK; Sampling: hand; individualCount: 2; **Location:** country: Russia; stateProvince: Krasnodar; locality: {11}; verbatimCoordinates: 44°42'56''N, 37°28'50'' E; 54; **Event:** eventDate: 06-15-13
- **Type status:** Other material. **Occurrence:** recordedBy: DMK; Sampling: hand; individualCount: 3; **Location:** country: Russia; stateProvince: Krasnodar; locality: {10}; verbatimCoordinates: 44°43'31''N, 37°29'04'' E; 85; **Event:** eventDate: 06-20-13
- **Type status:** Other material. **Occurrence:** recordedBy: IHT; Sampling: hand; individualCount: 2; **Location:** country: Russia; stateProvince: Krasnodar; locality: {2}; verbatimCoordinates: 44°44'13'' N, 37°28'46'' E; 153; **Event:** eventDate: 06/2013
- **Type status:** Other material. **Occurrence:** recordedBy: IHT; Sampling: hand; individualCount: 3; **Location:** country: Russia; stateProvince: Krasnodar; locality: {8}; verbatimCoordinates: 44°45'14'' N, 37°27'26'' E; 308; **Event:** eventDate: 06-19-13
- **Type status:** Other material. **Occurrence:** recordedBy: KBG, DIK, AAP, IHT; Sampling: hand, sample; individualCount: 5; **Location:** country: Russia; stateProvince: Krasnodar; locality: {1}; verbatimCoordinates: 44°45'15'' N, 37°29'53'' E; 195; **Event:** eventDate: 06/2013
- **Type status:** Other material. **Occurrence:** recordedBy: KBG, DIK, DMK, AAP, IHT; Sampling: hand, sample; individualCount: 1; **Location:** country: Russia; stateProvince: Krasnodar; locality: {2}; verbatimCoordinates: 44°44'13'' N, 37°28'46'' E; 153; **Event:** eventDate: 06/2013
- **Type status:** Other material. **Occurrence:** recordedBy: TYL; Sampling: Corer; individualCount: 4; **Location:** country: Russia; stateProvince: Krasnodar; locality: {10}; verbatimCoordinates: 44°43'31''N, 37°29'04'' E; 85; **Event:** eventDate: 06/2011
- **Type status:** Other material. **Occurrence:** recordedBy: TYL; Sampling: Corer; individualCount: 1; **Location:** country: Russia; stateProvince: Krasnodar; locality: *C. orientalis -Q. pubescens* forest with *T. begoniifolia* and *F. excelsior*; **Event:** eventDate: 06/2011
- **Type status:** Other material. **Occurrence:** recordedBy: TYL; Sampling: Corer; individualCount: 1; **Location:** country: Russia; stateProvince: Krasnodar; locality: *Q. pubescens - J. excelsa* forest; **Event:** eventDate: 06/2011

##### Notes

*C. caucasicus* is a species with the Caucasian and eastern Anatolian distribution (Bonato et al. 2011). The species was found in compressed fermentation-humus (FH) layer and soil.

#### Clinopodes escherichii

(Verhoeff, 1896)

##### Materials

- **Type status:** Other material. **Occurrence:** recordedBy: DIK, AAP, IHT; Sampling: hand; sample; individualCount: 15; **Location:** country: Russia; stateProvince: Krasnodar; locality: {4}; verbatimCoordinates: 44°42'50'' N, 37°27'01'' E; 8; **Event:** eventDate: 06/2013
- **Type status:** Other material. **Occurrence:** recordedBy: DMK; Sampling: hand; individualCount: 9; **Location:** country: Russia; stateProvince: Krasnodar; locality: {10}; verbatimCoordinates: 44°43'31''N, 37°29'04'' E; 85; **Event:** eventDate: 06-20-12
- **Type status:** Other material. **Occurrence:** recordedBy: IHT; Sampling: hand; individualCount: 1; **Location:** country: Russia; stateProvince: Krasnodar; locality: {2}; verbatimCoordinates: 44°44'13'' N, 37°28'46'' E; 153; **Event:** eventDate: 06/2013
- **Type status:** Other material. **Occurrence:** recordedBy: IHT; Sampling: hand; individualCount: 4; **Location:** country: Russia; stateProvince: Krasnodar; locality: {13}; verbatimCoordinates: 44°42'39''N, 37°28'37'' E; 31; **Event:** eventDate: 06-18-13
- **Type status:** Other material. **Occurrence:** recordedBy: IHT; Sampling: hand; individualCount: 3; **Location:** country: Russia; stateProvince: Krasnodar; locality: *P. mutica - Juniperus* shrubland on the dry slope; verbatimCoordinates: 44°42'48'' N, 37°27'58'' E; 126; **Event:** eventDate: 06-18-13
- **Type status:** Other material. **Occurrence:** recordedBy: IHT; Sampling: hand; individualCount: 2; **Location:** country: Russia; stateProvince: Krasnodar; locality: {8}; verbatimCoordinates: 44°45'14'' N, 37°27'26'' E; 308; **Event:** eventDate: 06-19-13
- **Type status:** Other material. **Occurrence:** recordedBy: KBG, DIK; Sampling: sample; individualCount: 2; **Location:** country: Russia; stateProvince: Krasnodar; locality: {5}; verbatimCoordinates: 44°42'38'' N, 37°27'31'' E; 10; **Event:** eventDate: 06/2009
- **Type status:** Other material. **Occurrence:** recordedBy: KBG, DIK; Sampling: sample; individualCount: 1; **Location:** country: Russia; stateProvince: Krasnodar; locality: {7}; verbatimCoordinates: 44°42'29'', 37°27'28'' E; 2; **Event:** eventDate: 06/2009
- **Type status:** Other material. **Occurrence:** recordedBy: KBG, DIK, AAP, IHT; Sampling: hand, sample; individualCount: 4; **Location:** country: Russia; stateProvince: Krasnodar; locality: {1}; verbatimCoordinates: 44°45'15'' N, 37°29'53'' E; 195; **Event:** eventDate: 06/2013
- **Type status:** Other material. **Occurrence:** recordedBy: KBG, DIK, DMK, AAP, IHT; Sampling: hand, sample; individualCount: 2; **Location:** country: Russia; stateProvince: Krasnodar; locality: {11}; verbatimCoordinates: 44°42'56''N, 37°28'50'' E; 54; **Event:** eventDate: 06-15-12
- **Type status:** Other material. **Occurrence:** recordedBy: KBG, DIK, DMK, AAP, IHT; Sampling: hand, sample; individualCount: 3; **Location:** country: Russia; stateProvince: Krasnodar; locality: {12}; verbatimCoordinates: 44°42'48''N, 37°28'39'' E; 50; **Event:** eventDate: 06-16-12
- **Type status:** Other material. **Occurrence:** recordedBy: KBG, DIK, DMK, AAP, IHT; Sampling: hand, sample; individualCount: 3; **Location:** country: Russia; stateProvince: Krasnodar; locality: {13}; verbatimCoordinates: 44°42'39''N, 37°28'37'' E; 31; **Event:** eventDate: 06-17-12
- **Type status:** Other material. **Occurrence:** recordedBy: KBG, DIK, DMK, AAP, IHT; Sampling: hand, sample; individualCount: 7; **Location:** country: Russia; stateProvince: Krasnodar; locality: {3}; verbatimCoordinates: 44°42'51'' N, 37°28'45'' E; 47; **Event:** eventDate: 06/2013
- **Type status:** Other material. **Occurrence:** recordedBy: TYL; Sampling: Corer; individualCount: 4; **Location:** country: Russia; stateProvince: Krasnodar; locality: *Q. pubescens - C. orientalis* forest; **Event:** eventDate: 06-08-10
- **Type status:** Other material. **Occurrence:** recordedBy: TYL; Sampling: Corer; individualCount: 1; **Location:** country: Russia; stateProvince: Krasnodar; locality: *C. orientalis* forest with *F. excelsior, Q. pubescens* and *T. begoniifolia*; **Event:** eventDate: 06-09-10
- **Type status:** Other material. **Occurrence:** recordedBy: TYL; Sampling: Corer; individualCount: 1; **Location:** country: Russia; stateProvince: Krasnodar; locality: *Q. pubescens - C. orientalis* forest; verbatimCoordinates: 44°43'41 N, 37°29'25'' E; **Event:** eventDate: 06-09-10
- **Type status:** Other material. **Occurrence:** recordedBy: TYL; Sampling: Corer; individualCount: 2; **Location:** country: Russia; stateProvince: Krasnodar; locality: *C. orientalis - Q. pubescens* forest with *T. begoniifolia* and *F. excelsior*; **Event:** eventDate: 06-13-10
- **Type status:** Other material. **Occurrence:** recordedBy: TYL; Sampling: Corer; individualCount: 1; **Location:** country: Russia; stateProvince: Krasnodar; locality: *Q. pubescens - Pinus pityusa* forest; **Event:** eventDate: 06-13-10
- **Type status:** Other material. **Occurrence:** recordedBy: TYL; Sampling: Corer; individualCount: 2; **Location:** country: Russia; stateProvince: Krasnodar; locality: *Juniperus - Quercus* shrubland, with *C. orientalis*; **Event:** eventDate: 06-14-10
- **Type status:** Other material. **Occurrence:** recordedBy: TYL; Sampling: Corer; individualCount: 2; **Location:** country: Russia; stateProvince: Krasnodar; locality: Woodless locality north of the Lake Sukhoy Liman; **Event:** eventDate: 06-14-10
- **Type status:** Other material. **Occurrence:** recordedBy: TYL; Sampling: Corer; individualCount: 5; **Location:** country: Russia; stateProvince: Krasnodar; locality: *Q. petraea - F. excelsior* forest; **Event:** eventDate: 06-17-10
- **Type status:** Other material. **Occurrence:** recordedBy: TYL; Sampling: Corer; individualCount: 2; **Location:** country: Russia; stateProvince: Krasnodar; locality: *Q. pubescens-C. orientalis* forest; **Event:** eventDate: 06-17-10
- **Type status:** Other material. **Occurrence:** recordedBy: TYL; Sampling: Corer; individualCount: 1; **Location:** country: Russia; stateProvince: Krasnodar; locality: Tangle of *P. spina-christi*; **Event:** eventDate: 06-18-10
- **Type status:** Other material. **Occurrence:** recordedBy: TYL; Sampling: Corer; individualCount: 2; **Location:** country: Russia; stateProvince: Krasnodar; locality: {10}; verbatimCoordinates: 44°43'31''N, 37°29'04'' E; 85; **Event:** eventDate: 06/2011
- **Type status:** Other material. **Occurrence:** recordedBy: TYL; Sampling: Corer; individualCount: 12; **Location:** country: Russia; stateProvince: Krasnodar; locality: *A. laetum - F. excelsior* forest with *Q. petraea*; **Event:** eventDate: 06/2011
- **Type status:** Other material. **Occurrence:** recordedBy: TYL; Sampling: Corer; individualCount: 2; **Location:** country: Russia; stateProvince: Krasnodar; locality: *F. orientalis* forest with *C. caucasica*; **Event:** eventDate: 06/2011
- **Type status:** Other material. **Occurrence:** recordedBy: TYL; Sampling: Corer; individualCount: 2; **Location:** country: Russia; stateProvince: Krasnodar; locality: *Q. pubescens - C. orientalis* forest; **Event:** eventDate: 06/2011
- **Type status:** Other material. **Occurrence:** recordedBy: TYL; Sampling: Corer; individualCount: 7; **Location:** country: Russia; stateProvince: Krasnodar; locality: *T. begoniifolia - Q. petraea* forest; **Event:** eventDate: 06/2011

##### Notes

This species inhabits soil and litter layers of various forests in Russia and Ukraine surrounding the Black Sea. It is also known to occur in the Balkans and extends to the Carpathians in the north. Several records are known from some Aegean islands and Anatolia (Bonato et al. 2011).

#### Diphyonyx conjungens

(Verhoeff, 1898)

##### Materials

- **Type status:** Other material. **Occurrence:** recordedBy: DMK; Sampling: hand; individualCount: 3; **Location:** country: Russia; stateProvince: Krasnodar; locality: {10}; verbatimCoordinates: 44°43'31''N, 37°29'04'' E; 85; **Event:** eventDate: 06-14-11
- **Type status:** Other material. **Occurrence:** recordedBy: DMK; Sampling: hand; individualCount: 2; **Location:** country: Russia; stateProvince: Krasnodar; locality: {10}; verbatimCoordinates: 44°43'31''N, 37°29'04'' E; 85; **Event:** eventDate: 06-20-12
- **Type status:** Other material. **Occurrence:** recordedBy: IHT; Sampling: hand; individualCount: 2; **Location:** country: Russia; stateProvince: Krasnodar; locality: {14}; verbatimCoordinates: 44°42'21'' N, 37°28'15'' E; 16; **Event:** eventDate: 06/2013
- **Type status:** Other material. **Occurrence:** recordedBy: KBG, DIK, AAP, IHT; Sampling: hand, sample; individualCount: 5; **Location:** country: Russia; stateProvince: Krasnodar; locality: {1}; verbatimCoordinates: 44°45'15'' N, 37°29'53'' E; 195; **Event:** eventDate: 06/2013
- **Type status:** Other material. **Occurrence:** recordedBy: KBG, DIK, DMK, AAP, IHT; Sampling: hand, sample; individualCount: 1; **Location:** country: Russia; stateProvince: Krasnodar; locality: {2}; verbatimCoordinates: 44°44'13'' N, 37°28'46'' E; 153; **Event:** eventDate: 06/2013
- **Type status:** Other material. **Occurrence:** recordedBy: TYL; Sampling: Corer; individualCount: 1; **Location:** country: Russia; stateProvince: Krasnodar; locality: *C. orientalis - Q. pubescens* forest with *T. begoniifolia* and *F. excelsior*; **Event:** eventDate: 06-13-10
- **Type status:** Other material. **Occurrence:** recordedBy: TYL; Sampling: Corer; individualCount: 2; **Location:** country: Russia; stateProvince: Krasnodar; locality: {10}; verbatimCoordinates: 44°43'31''N, 37°29'04'' E; 85; **Event:** eventDate: 06/2011
- **Type status:** Other material. **Occurrence:** recordedBy: TYL; Sampling: Corer; individualCount: 2; **Location:** country: Russia; stateProvince: Krasnodar; locality: *F. orientalis* forest with *C. caucasica*; **Event:** eventDate: 06/2011
- **Type status:** Other material. **Occurrence:** recordedBy: TYL; Sampling: Corer; individualCount: 2; **Location:** country: Russia; stateProvince: Krasnodar; locality: *T. begoniifolia* - *Q. petraea* forest; **Event:** eventDate: 06/2011
- **Type status:** Other material. **Occurrence:** recordedBy: TYL; Sampling: Corer; individualCount: 1; **Location:** country: Russia; stateProvince: Krasnodar; locality: *Q. petraea, F. orientalis* forest with *T. begoniifolia*; **Event:** eventDate: 06-18-13

##### Notes

*D. conjungens* is found in the Crimea, and also recorded in the Balkan Peninsula, throughout the entire Anatolia from the western coast and southern Sporades islands to the easternmost part of Western Armenia, northwards to the Pontic mountains and southwards to the Tauric mountains (Bonato et al. 2008).

#### Geophilus oligopus

(Attems, 1895)

##### Materials

- **Type status:** Other material. **Occurrence:** recordedBy: TYL; Sampling: Corer; individualCount: 2; **Location:** country: Russia; stateProvince: Krasnodar; locality: *Juniperus*-*Quercus* shrubland, with *C. orientalis*; **Event:** eventDate: 06-16-10
- **Type status:** Other material. **Occurrence:** recordedBy: TYL; Sampling: Corer; individualCount: 4; **Location:** country: Russia; stateProvince: Krasnodar; locality: {10}; verbatimCoordinates: 44°43'31''N, 37°29'04'' E; 85; **Event:** eventDate: 06/2011
- **Type status:** Other material. **Occurrence:** recordedBy: TYL; Sampling: Corer; individualCount: 4; **Location:** country: Russia; stateProvince: Krasnodar; locality: *A. laetum* - *F. excelsior* forest with *Q. petraea*; **Event:** eventDate: 06/2011
- **Type status:** Other material. **Occurrence:** recordedBy: TYL; Sampling: Corer; individualCount: 3; **Location:** country: Russia; stateProvince: Krasnodar; locality: *F. orientalis* forest with *C. caucasica*; **Event:** eventDate: 06/2011
- **Type status:** Other material. **Occurrence:** recordedBy: TYL; Sampling: Corer; individualCount: 3; **Location:** country: Russia; stateProvince: Krasnodar; locality: *P. mutica - Juniperus* shrubland; verbatimCoordinates: 44°42'38'' N, 37°27'55'' E; 51; **Event:** eventDate: 06/2011
- **Type status:** Other material. **Occurrence:** recordedBy: TYL; Sampling: Corer; individualCount: 1; **Location:** country: Russia; stateProvince: Krasnodar; locality: *Q. pubescens - C. orientalis* forest; **Event:** eventDate: 06/2011
- **Type status:** Other material. **Occurrence:** recordedBy: TYL; Sampling: Corer; individualCount: 8; **Location:** country: Russia; stateProvince: Krasnodar; locality: *T. begoniifolia - Q. petraea* forest; **Event:** eventDate: 06/2011

##### Notes

In habitus, the studied material strongly resembles *G. oligopus*, although our localities are situated far away from the currently known range of the species. Until now, *G. oligopus* has mostly been known from the Alpine-Dinaric area, being also recorded in the Carpathians (Christian 1996, Dányi 2007). In the Abrau Peninsula, the specimens were found beneath stones.

#### Pachymerium ferrugineum

(C.L. Koch, 1835)

##### Materials

- **Type status:** Other material. **Occurrence:** recordedBy: IHT; Sampling: hand; individualCount: 3; **Location:** country: Russia; stateProvince: Krasnodar; locality: {2}; verbatimCoordinates: 44°44'13'' N, 37°28'46'' E; 153; **Event:** eventDate: 06/2013
- **Type status:** Other material. **Occurrence:** recordedBy: IHT; Sampling: hand; individualCount: 1; **Location:** country: Russia; stateProvince: Krasnodar; locality: {8}; verbatimCoordinates: 44°45'14'' N, 37°27'26'' E; 308; **Event:** eventDate: 06-19-13
- **Type status:** Other material. **Occurrence:** recordedBy: KBG, DIK, AAP, IHT; Sampling: hand, sample; individualCount: 6; **Location:** country: Russia; stateProvince: Krasnodar; locality: {1}; verbatimCoordinates: 44°45'15'' N, 37°29'53'' E; 195; **Event:** eventDate: 06/2013
- **Type status:** Other material. **Occurrence:** recordedBy: KBG, DIK, DMK, AAP, IHT; Sampling: hand, sample; individualCount: 4; **Location:** country: Russia; stateProvince: Krasnodar; locality: {2}; verbatimCoordinates: 44°44'13'' N, 37°28'46'' E; 153; **Event:** eventDate: 06/2013
- **Type status:** Other material. **Occurrence:** recordedBy: KBG, DIK, DMK, AAP, IHT; Sampling: hand, sample; individualCount: 5; **Location:** country: Russia; stateProvince: Krasnodar; locality: {3}; verbatimCoordinates: 44°42'51'' N, 37°28'45'' E; 47; **Event:** eventDate: 06/2013
- **Type status:** Other material. **Occurrence:** recordedBy: KBG, DIK, DMK, AAP, IHT; Sampling: hand, sample; individualCount: 1; **Location:** country: Russia; stateProvince: Krasnodar; locality: {11}; verbatimCoordinates: 44°42'56''N, 37°28'50'' E; 54; **Event:** eventDate: 06-15-13
- **Type status:** Other material. **Occurrence:** recordedBy: TYL; Sampling: Corer; individualCount: 1; **Location:** country: Russia; stateProvince: Krasnodar; locality: *C. caucasica - F. orientalis* forest; **Event:** eventDate: 06-11-10
- **Type status:** Other material. **Occurrence:** recordedBy: TYL; Sampling: Corer; individualCount: 3; **Location:** country: Russia; stateProvince: Krasnodar; locality: *Q. petraea, F. orientalis* forest with *T. begoniifolia*; **Event:** eventDate: 06-16-10
- **Type status:** Other material. **Occurrence:** recordedBy: TYL; Sampling: Corer; individualCount: 2; **Location:** country: Russia; stateProvince: Krasnodar; locality: {10}; verbatimCoordinates: 44°43'31''N, 37°29'04'' E; 85; **Event:** eventDate: 06/2011

##### Notes

*P. ferrugineum* is widely distributed through most parts of the Palaearctic (Palmén 1988, Simaiakis and Mylonas 2003). In Russia it is known from Southern to Far Eastern federal districts and from Murmansk region through Central Russia and Caucasus to Armenia. The species shows preference for various types of pine forests and for seashores, less frequently it is found in mixed and deciduous forests, steppes, meadows and agricultural areas (Zalesskaja et al. 1982). In the Abrau Peninsula it was mainly collected from the compressed FH layer and beneath the stones.

#### Schendylidae

#### Schendyla nemorensis

(C.L. Koch, 1837)

##### Materials

- **Type status:** Other material. **Occurrence:** recordedBy: TYL; Sampling: Corer; individualCount: 3; **Location:** country: Russia; stateProvince: Krasnodar; locality: Q. petraea, F. orientalis forest with T. begoniifolia; **Event:** eventDate: 06/2011
- **Type status:** Other material. **Occurrence:** recordedBy: TYL; Sampling: Corer; individualCount: 2; **Location:** country: Russia; stateProvince: Krasnodar; locality: *Q. pubescens - J. excelsa* forest; **Event:** eventDate: 06/2011

##### Notes

A common soil-dwelling European species, distributed from the Great Britain to Macaronesia and through the Mediterranean region to Rostov region of Russia (Zalesskaja et al. 1982, Bonato et al. 2005). *S. nemorensis* tends to occur in the soils of open areas, less frequently in coniferous and deciduous forests, steppes and agricultural landscapes (Zalesskaja et al. 1982). In the Abrau Peninsula, the representatives of the species were collected from the soil and beneath the stones.

#### Diplopoda

#### Polyxenida

#### Polyxenidae

#### Propolyxenus aegeus

(Silvestri, 1948) / P. trivittatus (Verhoeff , 1941)

##### Materials

- **Type status:** Other material. **Occurrence:** recordedBy: IHT; Sampling: hand, whipping; individualCount: 7; **Location:** country: Russia; stateProvince: Krasnodar; locality: {10}; verbatimCoordinates: 44°43'31''N, 37°29'04'' E; 85; **Event:** eventDate: 06-14-13
- **Type status:** Other material. **Occurrence:** recordedBy: KBG, DIK, DMK, AAP, IHT; Sampling: hand, sample, whipping; individualCount: 3; **Location:** country: Russia; stateProvince: Krasnodar; locality: {2}; verbatimCoordinates: 44°44'13'' N, 37°28'46'' E; 153; **Event:** eventDate: 06-15-13

##### Notes

*P. trivittatus* is known from Israel, western Turkey and Greece; *P. aegeus* is found in Rhodes (Short and Huynh 2010). The identification of these two species is extremely difficult. Due to their strikingly similar structural details, they could even be synonyms. The question of the taxonomic status of these two species remains open (Short and Huynh 2010). In the region, specimens were collected by way of whipping the branches of bushes and trees.

#### Lophoproctidae

#### Lophoproctus coecus

(Pocock, 1888)

##### Materials

- **Type status:** Other material. **Occurrence:** recordedBy: IIS; Sampling: Hand; individualCount: 11; **Location:** country: Russia; stateProvince: Krasnodar; locality: {17}; verbatimCoordinates: 44°43'46''N, 37°29'13'' E; 116; **Event:** eventDate: 06/2011
- **Type status:** Other material. **Occurrence:** recordedBy: KBG, DIK, AAP, IHT; Sampling: hand, sample; individualCount: 25; **Location:** country: Russia; stateProvince: Krasnodar; locality: {1}; verbatimCoordinates: 44°45'15'' N, 37°29'53'' E; 195; **Event:** eventDate: 06/2013
- **Type status:** Other material. **Occurrence:** recordedBy: KBG, DIK, DMK, AAP, IHT; Sampling: hand, sample; individualCount: 17; **Location:** country: Russia; stateProvince: Krasnodar; locality: {11}; verbatimCoordinates: 44°42'56''N, 37°28'50'' E; 54; **Event:** eventDate: 06/2013
- **Type status:** Other material. **Occurrence:** recordedBy: KBG, DIK, DMK, AAP, IHT; Sampling: hand, sample; individualCount: 3; **Location:** country: Russia; stateProvince: Krasnodar; locality: {13}; verbatimCoordinates: 44°42'39''N, 37°28'37'' E; 31; **Event:** eventDate: 06/2013
- **Type status:** Other material. **Occurrence:** recordedBy: TYL; Sampling: Corer; individualCount: 7; **Location:** country: Russia; stateProvince: Krasnodar; locality: Q. pubescens-Pinus pityusa forest; **Event:** eventDate: 06-13-10
- **Type status:** Other material. **Occurrence:** recordedBy: TYL; Sampling: Corer; individualCount: 21; **Location:** country: Russia; stateProvince: Krasnodar; locality: {10}; verbatimCoordinates: 44°43'31''N, 37°29'04'' E; 85; **Event:** eventDate: 06/2011
- **Type status:** Other material. **Occurrence:** recordedBy: TYL; Sampling: Corer; individualCount: 2; **Location:** country: Russia; stateProvince: Krasnodar; locality: F. orientalis forest with C. caucasica; **Event:** eventDate: 06/2011

##### Notes

*L. coecus* is widespread throughout Europe, particularly in Eastern Europe with its distribution extending to Central Asia (Short 2015). In Abrau Peninsula, the species was collected from the soil and litter layers of various forest types.

#### Glomerida

#### Glomeridae

#### Hyleoglomeris awchasica

(Brandt 1840)

##### Materials

- **Type status:** Other material. **Occurrence:** recordedBy: IIS; Sampling: Hand; individualCount: 7; **Location:** country: Russia; stateProvince: Krasnodar; locality: {16}; verbatimCoordinates: 44°44'27''N, 37°29'53'' E; 295; **Event:** eventDate: 06/2011

##### Notes

This species is known from Colchis, Georgian and Russian parts of the western Caucasus (Golovatch et al. 2006). In the study area the animals were found in leaf litter, upper soil layers and under fallen twigs.

#### Trachysphaera costata

(Waga, 1857)

##### Materials

- **Type status:** Other material. **Occurrence:** recordedBy: IIS; Sampling: Hand; individualCount: 20; **Location:** country: Russia; stateProvince: Krasnodar; locality: {16}; verbatimCoordinates: 44°44'27''N, 37°29'53'' E; 295; **Event:** eventDate: 06/2011
- **Type status:** Other material. **Occurrence:** recordedBy: IIS; Sampling: Hand; individualCount: 12; **Location:** country: Russia; stateProvince: Krasnodar; locality: {18}; verbatimCoordinates: 44°44'02''N, 37°29'32'' E; 172; **Event:** eventDate: 06/2011
- **Type status:** Other material. **Occurrence:** recordedBy: IIS; Sampling: Hand; individualCount: 13; **Location:** country: Russia; stateProvince: Krasnodar; locality: {19}; verbatimCoordinates: 44°44'10''N, 37°28'47'' E; 149; **Event:** eventDate: 06/2011
- **Type status:** Other material. **Occurrence:** recordedBy: IIS; Sampling: Hand; individualCount: 23; **Location:** country: Russia; stateProvince: Krasnodar; locality: {15}; verbatimCoordinates: 44°45'02''N, 37°30'05'' E; 273; **Event:** eventDate: 06/2013

##### Notes

*T. costata* is known from parthenogenetic populations in Central and Eastern Europe, as well as in some areas in the Caucasus, while bisexual populations are restricted to southern Romania, the Balkans, Anatolia, Israel, most of the Caucasus, and Crimea (Golovatch 2008, Golovatch 2012). Quite often specimens are found in caves (Golovatch 2008). In the Abrau Peninsula, the species was collected from the upper soil and lower leaf litter layers.

#### Polyzoniida

#### Hirudisomatidae

#### Hirudisoma roseum

(Victor, 1839)

##### Materials

- **Type status:** Other material. **Occurrence:** recordedBy: IIS; Sampling: Hand; individualCount: 6; **Location:** country: Russia; stateProvince: Krasnodar; locality: {15}; verbatimCoordinates: 44°45'02''N, 37°30'05'' E; 273; **Event:** eventDate: 06/2011
- **Type status:** Other material. **Occurrence:** recordedBy: IIS; Sampling: Hand; individualCount: 2; **Location:** country: Russia; stateProvince: Krasnodar; locality: {16}; verbatimCoordinates: 44°44'27''N, 37°29'53'' E; 295; **Event:** eventDate: 06/2011

##### Notes

The species distribution covers southern Russia, Abkhazia, Georgia, north-western Azerbaijan and eastern Turkey (Golovatch et al. 2015). *H. roseum* is known as a typical leaf litter dweller. In the Abrau Peninsula, some individuals were collected from the compressed FH layer densely colonized by saprotrophic mycelium. This may be due to the altered, siphon-like mouthparts of Hirudisomatidans, which are presumably used for uptaking fluid food (e.g. hyphal contents) (Read and Enghoff 2009).

#### Julida

#### Blaniulidae

#### Nopoiulus kochii

(Gervais, 1847)

##### Materials

- **Type status:** Other material. **Occurrence:** recordedBy: IIS; Sampling: Hand; individualCount: 4; **Location:** country: Russia; stateProvince: Krasnodar; locality: {15} under bark of fallen branches; verbatimCoordinates: 44°45'02''N, 37°30'05'' E; 273; **Event:** eventDate: 06/2011
- **Type status:** Other material. **Occurrence:** recordedBy: IIS; Sampling: Hand; individualCount: 7; **Location:** country: Russia; stateProvince: Krasnodar; locality: {17}; verbatimCoordinates: 44°43'46''N, 37°29'13'' E; 116; **Event:** eventDate: 06/2011
- **Type status:** Other material. **Occurrence:** recordedBy: IIS; Sampling: Hand; individualCount: 9; **Location:** country: Russia; stateProvince: Krasnodar; locality: {18}; verbatimCoordinates: 44°44'02''N, 37°29'32'' E; 172; **Event:** eventDate: 06/2011

##### Notes

*N. kochii* is recorded widely across the continent. It has been introduced to New Zealand, North and South America (Enghoff and Kime 2005). The habitats of *Nopoiulus* species are closely related to decaying wood. In the area, some individuals were collected from leaf litter in broad-leaf forests.

#### Nemasomatidae

#### Nemasoma caucasicum

(Lohmander, 1932)

##### Materials

- **Type status:** Other material. **Occurrence:** recordedBy: IIS; Sampling: Hand; individualCount: 3; **Location:** country: Russia; stateProvince: Krasnodar; locality: {15}; verbatimCoordinates: 44°45'02''N, 37°30'05'' E; 273; **Event:** eventDate: 06/2011
- **Type status:** Other material. **Occurrence:** recordedBy: IIS; Sampling: Hand; individualCount: 3; **Location:** country: Russia; stateProvince: Krasnodar; locality: {16}; verbatimCoordinates: 44°44'27''N, 37°29'53'' E; 295; **Event:** eventDate: 06/2011
- **Type status:** Other material. **Occurrence:** recordedBy: IIS; Sampling: Hand; individualCount: 1; **Location:** country: Russia; stateProvince: Krasnodar; locality: {17}; verbatimCoordinates: 44°43'46''N, 37°29'13'' E; 116; **Event:** eventDate: 06/2011

##### Notes

The species is known from Turkey, Armenia, Azerbaidjan, Georgia and southern Russia (Jensen et al. 2002, Enghoff 2006). In the area, it is strictly associated with decaying wood of fallen trees.

#### Julidae

#### Chaetoleptophyllum flexum

Golovatch, 1979

##### Materials

- **Type status:** Other material. **Occurrence:** recordedBy: IIS; Sampling: Hand; individualCount: 4; **Location:** country: Russia; stateProvince: Krasnodar; locality: {15}; verbatimCoordinates: 44°45'02''N, 37°30'05'' E; 273; **Event:** eventDate: 06/2011
- **Type status:** Other material. **Occurrence:** recordedBy: IIS; Sampling: Hand; individualCount: 10; **Location:** country: Russia; stateProvince: Krasnodar; locality: {17}; verbatimCoordinates: 44°43'46''N, 37°29'13'' E; 116; **Event:** eventDate: 06/2011
- **Type status:** Other material. **Occurrence:** recordedBy: IIS; Sampling: Hand; individualCount: 1; **Location:** country: Russia; stateProvince: Krasnodar; locality: {18}; verbatimCoordinates: 44°44'02''N, 37°29'32'' E; 172; **Event:** eventDate: 06/2011
- **Type status:** Other material. **Occurrence:** recordedBy: IIS; Sampling: Hand; individualCount: 1; **Location:** country: Russia; stateProvince: Krasnodar; locality: {19}; verbatimCoordinates: 44°44'10''N, 37°28'47'' E; 149; **Event:** eventDate: 06/2011

##### Notes

Identification of the above species was difficult as only females and juveniles were found. *C. flexum* is a common and abundant species in the Krasnodar region (Chumachenko, in litt.), it was also reported from Georgia, Stavropol region and the montane parts of the Republic of Adygea, Russia (Zuev 2014). In teh study area the specimens were collected from the thick layer of leaf litter, also from overmoistening sites, rarely under barks of fallen trees.

#### Cylindroiulus sp.

##### Materials

- **Type status:** Other material. **Occurrence:** recordedBy: IIS; Sampling: Hand; individualCount: 15; **Location:** country: Russia; stateProvince: Krasnodar; locality: {15}; verbatimCoordinates: 44°45'02''N, 37°30'05'' E; 273; **Event:** eventDate: 06/2011
- **Type status:** Other material. **Occurrence:** recordedBy: IIS; Sampling: Hand; individualCount: 15; **Location:** country: Russia; stateProvince: Krasnodar; locality: {16}; verbatimCoordinates: 44°44'27''N, 37°29'53'' E; 295; **Event:** eventDate: 06/2011
- **Type status:** Other material. **Occurrence:** recordedBy: IIS; Sampling: Hand; individualCount: 1; **Location:** country: Russia; stateProvince: Krasnodar; locality: {17}; verbatimCoordinates: 44°43'46''N, 37°29'13'' E; 116; **Event:** eventDate: 06/2011
- **Type status:** Other material. **Occurrence:** recordedBy: IIS; Sampling: Hand; individualCount: 13; **Location:** country: Russia; stateProvince: Krasnodar; locality: {18}; verbatimCoordinates: 44°44'02''N, 37°29'32'' E; 172; **Event:** eventDate: 06/2011
- **Type status:** Other material. **Occurrence:** recordedBy: IIS; Sampling: Hand; individualCount: 16; **Location:** country: Russia; stateProvince: Krasnodar; locality: {19}; verbatimCoordinates: 44°44'10''N, 37°28'47'' E; 149; **Event:** eventDate: 06/2011

##### Notes

This is most likely a new species that requires description. All specimens were collected from the leaf litter and under barks of fallen trees in all types of broad-leaf forests.

#### Julus colchicus

Lohmander, 1936

##### Materials

- **Type status:** Other material. **Occurrence:** recordedBy: IIS; Sampling: Hand; individualCount: 12; **Location:** country: Russia; stateProvince: Krasnodar; locality: {15}; verbatimCoordinates: 44°45'02''N, 37°30'05'' E; 273; **Event:** eventDate: 06/2011
- **Type status:** Other material. **Occurrence:** recordedBy: IIS; Sampling: Hand; individualCount: 2; **Location:** country: Russia; stateProvince: Krasnodar; locality: {16}; verbatimCoordinates: 44°44'27''N, 37°29'53'' E; 295; **Event:** eventDate: 06/2011
- **Type status:** Other material. **Occurrence:** recordedBy: IIS; Sampling: Hand; individualCount: 3; **Location:** country: Russia; stateProvince: Krasnodar; locality: {17}; verbatimCoordinates: 44°43'46''N, 37°29'13'' E; 116; **Event:** eventDate: 06/2011
- **Type status:** Other material. **Occurrence:** recordedBy: IIS; Sampling: Hand; individualCount: 4; **Location:** country: Russia; stateProvince: Krasnodar; locality: {18}; verbatimCoordinates: 44°44'02''N, 37°29'32'' E; 172; **Event:** eventDate: 06/2011
- **Type status:** Other material. **Occurrence:** recordedBy: IIS; Sampling: Hand; individualCount: 3; **Location:** country: Russia; stateProvince: Krasnodar; locality: {19}; verbatimCoordinates: 44°44'10''N, 37°28'47'' E; 149; **Event:** eventDate: 06/2011

##### Notes

*J. colchicus* is known from Turkey, Georgia, Abkhazia and the southern regions of Russia (Enghoff 2006, Zuev 2014). The specimens were collected from the leaf litter of broad-leaf forests.

#### Megaphyllum rossicum

(Timotheew, 1897)

##### Materials

- **Type status:** Other material. **Occurrence:** recordedBy: IIS; Sampling: Hand; individualCount: 1; **Location:** country: Russia; stateProvince: Krasnodar; locality: {17}; verbatimCoordinates: 44°43'46''N, 37°29'13'' E; 116; **Event:** eventDate: 06/2011
- **Type status:** Other material. **Occurrence:** recordedBy: IIS; Sampling: Hand; individualCount: 2; **Location:** country: Russia; stateProvince: Krasnodar; locality: {19}; verbatimCoordinates: 44°44'10''N, 37°28'47'' E; 149; **Event:** eventDate: 06/2011

##### Notes

*M. rossicum* is known from the south and center of European Russia, Crimea and the Caucasus, reaching Ural Mountains in the East (Golovatch 1992). In the region, the species was collected from the upper leaf litter layers.

#### Pachyiulus krivolutskyi

Golovatch, 1977

##### Materials

- **Type status:** Other material. **Occurrence:** recordedBy: IIS; Sampling: Hand; individualCount: 8; **Location:** country: Russia; stateProvince: Krasnodar; locality: {15}; verbatimCoordinates: 44°45'02''N, 37°30'05'' E; 273; **Event:** eventDate: 06/2011
- **Type status:** Other material. **Occurrence:** recordedBy: IIS; Sampling: Hand; individualCount: 3; **Location:** country: Russia; stateProvince: Krasnodar; locality: {16}; verbatimCoordinates: 44°44'27''N, 37°29'53'' E; 295; **Event:** eventDate: 06/2011
- **Type status:** Other material. **Occurrence:** recordedBy: IIS; Sampling: Hand; individualCount: 2; **Location:** country: Russia; stateProvince: Krasnodar; locality: {17}; verbatimCoordinates: 44°43'46''N, 37°29'13'' E; 116; **Event:** eventDate: 06/2011
- **Type status:** Other material. **Occurrence:** recordedBy: IIS; Sampling: Hand; individualCount: 1; **Location:** country: Russia; stateProvince: Krasnodar; locality: {18}; verbatimCoordinates: 44°44'02''N, 37°29'32'' E; 172; **Event:** eventDate: 06/2011
- **Type status:** Other material. **Occurrence:** recordedBy: IIS; Sampling: Hand; individualCount: 1; **Location:** country: Russia; stateProvince: Krasnodar; locality: {19}; verbatimCoordinates: 44°44'10''N, 37°28'47'' E; 149; **Event:** eventDate: 06/2011

##### Notes

*P. krivolutskyi* is mostly known from the Caucasus (Mauries et al. 1997, Mikhaljova and Golovatch 2004). In the study area, this species was collected from the deciduous forests with thick, fairly moist litter, where it builds burrows laid with dung.

#### Chordeumatida

#### Anthroleucosomatidae

#### gen. sp. undetermined

##### Materials

- **Type status:** Other material. **Occurrence:** recordedBy: IIS; Sampling: Hand; individualCount: 1; **Location:** country: Russia; stateProvince: Krasnodar; locality: {15}; verbatimCoordinates: 44°45'02''N, 37°30'05'' E; 273; **Event:** eventDate: 06/2011
- **Type status:** Other material. **Occurrence:** recordedBy: IIS; Sampling: Hand; individualCount: 5; **Location:** country: Russia; stateProvince: Krasnodar; locality: {16}; verbatimCoordinates: 44°44'27''N, 37°29'53'' E; 295; **Event:** eventDate: 06/2011
- **Type status:** Other material. **Occurrence:** recordedBy: IIS; Sampling: Hand; individualCount: 3; **Location:** country: Russia; stateProvince: Krasnodar; locality: {17}; verbatimCoordinates: 44°43'46''N, 37°29'13'' E; 116; **Event:** eventDate: 06/2011
- **Type status:** Other material. **Occurrence:** recordedBy: IIS; Sampling: Hand; individualCount: 2; **Location:** country: Russia; stateProvince: Krasnodar; locality: {18}; verbatimCoordinates: 44°44'02''N, 37°29'32'' E; 172; **Event:** eventDate: 06/2011

##### Notes

This is most likely a new, yet undescribed species. The specimens were found in broad-leaf forests, under the bark of fallen trees and branches, rarely in leaf litter.

#### Polydesmida

#### Polydesmidae

#### Brachydesmus furcatus

Lohmander, 1936

##### Materials

- **Type status:** Other material. **Occurrence:** recordedBy: IIS; Sampling: Hand; individualCount: 4; **Location:** country: Russia; stateProvince: Krasnodar; locality: {16}; verbatimCoordinates: 44°44'27''N, 37°29'53'' E; 295; **Event:** eventDate: 06/2011
- **Type status:** Other material. **Occurrence:** recordedBy: IIS; Sampling: Hand; individualCount: 2; **Location:** country: Russia; stateProvince: Krasnodar; locality: {17}; verbatimCoordinates: 44°43'46''N, 37°29'13'' E; 116; **Event:** eventDate: 06/2011

##### Notes

*B. furcatus* was described in the Northern Caucasus (Lohmander 1936), but the exact range of the species remains unknown. In the studied region, it occurs in broad-leaf (*T. begoniifolia* and *F. orientalis*) forests, mainly in the upper soil and the compressed FH layers.

#### Brachydesmus kalischewskyi

Lignau, 1915

##### Materials

- **Type status:** Other material. **Occurrence:** recordedBy: IIS; Sampling: Hand; individualCount: 2; **Location:** country: Russia; stateProvince: Krasnodar; locality: {15}; verbatimCoordinates: 44°45'02''N, 37°30'05'' E; 273; **Event:** eventDate: 06/2011
- **Type status:** Other material. **Occurrence:** recordedBy: IIS; Sampling: Hand; individualCount: 5; **Location:** country: Russia; stateProvince: Krasnodar; locality: {17}; verbatimCoordinates: 44°43'46''N, 37°29'13'' E; 116; **Event:** eventDate: 06/2011
- **Type status:** Other material. **Occurrence:** recordedBy: IIS; Sampling: Hand; individualCount: 3; **Location:** country: Russia; stateProvince: Krasnodar; locality: {18}; verbatimCoordinates: 44°44'02''N, 37°29'32'' E; 172; **Event:** eventDate: 06/2011

##### Notes

The species is known from the western part of Turkey, Adsharia and Abkhasia (Enghoff 2006). In the Abrau Peninsula, it occurs in compressed FH layer of broad-leaf forests.

#### Polydesmus muralewiczi

Lohmander, 1936

##### Materials

- **Type status:** Other material. **Occurrence:** recordedBy: IIS; Sampling: Hand; individualCount: 13; **Location:** country: Russia; stateProvince: Krasnodar; locality: {15}; verbatimCoordinates: 44°45'02''N, 37°30'05'' E; 273; **Event:** eventDate: 06/2011
- **Type status:** Other material. **Occurrence:** recordedBy: IIS; Sampling: Hand; individualCount: 19; **Location:** country: Russia; stateProvince: Krasnodar; locality: {16}; verbatimCoordinates: 44°44'27''N, 37°29'53'' E; 295; **Event:** eventDate: 06/2011
- **Type status:** Other material. **Occurrence:** recordedBy: IIS; Sampling: Hand; individualCount: 16; **Location:** country: Russia; stateProvince: Krasnodar; locality: {17}; verbatimCoordinates: 44°43'46''N, 37°29'13'' E; 116; **Event:** eventDate: 06/2011
- **Type status:** Other material. **Occurrence:** recordedBy: IIS; Sampling: Hand; individualCount: 9; **Location:** country: Russia; stateProvince: Krasnodar; locality: {18}; verbatimCoordinates: 44°44'02''N, 37°29'32'' E; 172; **Event:** eventDate: 06/2011
- **Type status:** Other material. **Occurrence:** recordedBy: IIS; Sampling: Hand; individualCount: 26; **Location:** country: Russia; stateProvince: Krasnodar; locality: {19}; verbatimCoordinates: 44°44'10''N, 37°28'47'' E; 149; **Event:** eventDate: 06/2011

##### Notes

The species is widespread in the Caucasus (Stavropol and Krasnodar regions of Russia and Western Georgia), where it occurs in a wide range of habitats (Zuev 2014). In the Abrau Peninsula, it is frequently found in leaf litter, rarely beneath the stones, and under the bark of fallen trees.

## Analysis

In total, in the Abrau Peninsula 33 species of Chilopoda and Diplopoda were collected. The centipede fauna is represented by typical Caucasian (e.g. *H. spinipes*, *C. caucasicus)* as well as Mediterranean elements (*D. conjungens*, *S. cingulata*) and widespread Palearctic species (e.g. *L. curtipes*, *P. ferrugineum*). The millipede fauna consists mainly of Caucasian (e.g. *B. furcatus, H. roseum, P. krivolutskyi*) and some Euromediterranean species (*M. rossicum, T. costata*). The highest species diversity of Diplopoda is noted in the mountainous deciduous forests (all 16 species). However, in xerophytic forests presence of typically Mediterranean species is also notable (*P. aegeus* / *P. trivittatus*).

The PCA (Fig. 2) clearly showed specificity of ecological preferences of species belonging to different geographic distribution patterns. Mediterranean species strongly and positively correlated with meso-xerophytic and to a smaller extent xerophytic habitats (e.g. xerophytic forests and shrublands with pistachio, juniper and oriental hornbeam). Some typically Mediterranean species, e.g. *S. cingulata* and actively mobile aboveground *S. coleoptrata* tended to inhabit xerophytic habitats. It is worth noting that species like *L. forficatus* and *L. curtipes* are also found in the Abrau peninsula only in the most xeric habitats. Thus, ecological conditions of xeric habitats "weed out" (sensu Siepel 1996) the species with local ranges, but allow the existence of eurytopic species with global distribution patterns. Centralasiatic species clearly preferred grassland habitats such as hilltops of piedmonts, southern slopes of Navagir Ridge (e.g. open areas near Sukhoy Liman Lake), dominated by typical steppe vegetation (e.g. *Stipa* spp.) (Fig. 2). The analysis further revealed that species showing the Caucasian distribution pattern are antagonistic to meso-xerophytic and xerophytic habitats, while European species show weak relationship with any kind of habitats. Palearctic species were strongly related with mesophylic and steppe habitats. Ecological conditions of mesophytic forests, are suitable for non-indigenous species, widely distributed in the northern hemisphere (e.g., all Palearctic and pan-Holartic). We can conclude that from the faunistic point of view meso-xerophytic and xerophytic habitats are similar and independent of the species composition in the steppic and mesophylic habitats, while species composition in the latter two habitat types is clearly antagonistic.

**Figure 3.**
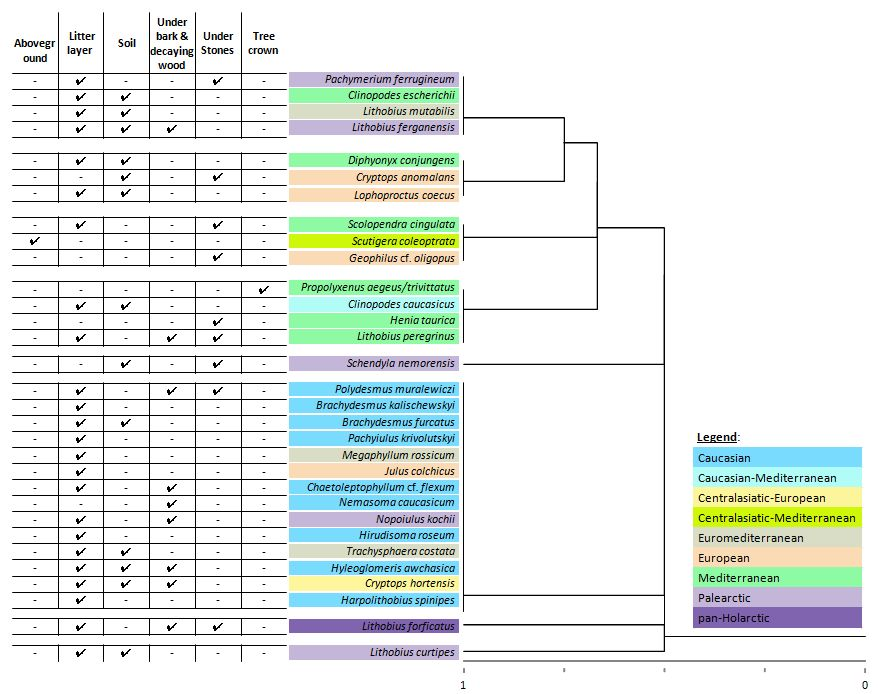
Cluster analysis of similarity (Jaccard) of myriapod species distribution patterns across sampling plots in the Abrau Peninsula with respect to their distribution type and microbiotope preferences. The geographical distribution pattern of each species has been marked using color-key. The table to the left of the cluster tree demonstrates microbiotopes, in which the respective species were found.

The cluster analysis revealed that habitat preferences of myriapods within the set of sampling plots (Table 1 and section "materials" in the checklist) are generally driven by species geographic distribution pattern and microbiotope preferences (Fig. 3). The species comprizing the largest cluster mainly belong to millipedes with Caucasian distribution (64%) which are strongly associated with thick leaf litter (Fig. 3). Such litter layer is typical for mesophytic forests. Mediterranean species prefer microbiotopes beneath the stones and lying tree trunks which are typical for meso-xerophytic forests characterized by thin litter layer (Table 1).

The top two clusters in Fig. 3 consist mainly of centipedes (except for *L. coecus*) with the wide geographic distribution. Their relative ecological tolerance to abiotic conditions at the microbiotope level allow them to occupy not only litter but also soil horizon (Fig. 3) and thus be equally frequent in both xerophytic shrublands (soil) and mesophytic beech forest (thick litter layer).

Our results suggest that the Abrau Peninsula is identified as a unique vegetation formation (Seregin and Suslova 2007). This is also true for the myriapod fauna. A range of ecological conditions on a relatively small peninsula allows the existence of a wide spectrum of myriapod species. This emphasizes the importance of biogeographic position of Abrau Peninsula and indicates some past connections between the northwestern Caucasus, the western part of Mediterranean region and Pontic steppes. Unfortunately, once widespread pristine habitats became very fragmented, but are still represented in Abrau Peninsula. The research conducted is the first step in a detailed study of the Myriapoda fauna of northwestern Caucasus, which should be continued.

## Supplementary Material

XML Treatment for Chilopoda

XML Treatment for Scutigeromorpha

XML Treatment for Scutigeridae

XML Treatment for Scutigera coleoptrata

XML Treatment for Lithobiomorpha

XML Treatment for Lithobiidae

XML Treatment for Harpolithobius spinipes

XML Treatment for Lithobius (Monotarsobius) curtipes

XML Treatment for Lithobius (Monotarsobius) ferganensis

XML Treatment for Lithobius forficatus

XML Treatment for Lithobius mutabilis

XML Treatment for Lithobius peregrinus

XML Treatment for Scolopendromorpha

XML Treatment for Cryptopidae

XML Treatment for Cryptops anomalans

XML Treatment for Cryptops hortensis

XML Treatment for Scolopendridae

XML Treatment for Scolopendra cingulata

XML Treatment for Geophilomorpha

XML Treatment for Dignathodontidae

XML Treatment for Henia (Meinertia) taurica

XML Treatment for Geophilidae

XML Treatment for Clinopodes caucasicus

XML Treatment for Clinopodes escherichii

XML Treatment for Diphyonyx conjungens

XML Treatment for Geophilus oligopus

XML Treatment for Pachymerium ferrugineum

XML Treatment for Schendylidae

XML Treatment for Schendyla nemorensis

XML Treatment for Diplopoda

XML Treatment for Polyxenida

XML Treatment for Polyxenidae

XML Treatment for Propolyxenus aegeus

XML Treatment for Lophoproctidae

XML Treatment for Lophoproctus coecus

XML Treatment for Glomerida

XML Treatment for Glomeridae

XML Treatment for Hyleoglomeris awchasica

XML Treatment for Trachysphaera costata

XML Treatment for Polyzoniida

XML Treatment for Hirudisomatidae

XML Treatment for Hirudisoma roseum

XML Treatment for Julida

XML Treatment for Blaniulidae

XML Treatment for Nopoiulus kochii

XML Treatment for Nemasomatidae

XML Treatment for Nemasoma caucasicum

XML Treatment for Julidae

XML Treatment for Chaetoleptophyllum flexum

XML Treatment for Cylindroiulus sp.

XML Treatment for Julus colchicus

XML Treatment for Megaphyllum rossicum

XML Treatment for Pachyiulus krivolutskyi

XML Treatment for Chordeumatida

XML Treatment for Anthroleucosomatidae

XML Treatment for gen. sp. undetermined

XML Treatment for Polydesmida

XML Treatment for Polydesmidae

XML Treatment for Brachydesmus furcatus

XML Treatment for Brachydesmus kalischewskyi

XML Treatment for Polydesmus muralewiczi

## Figures and Tables

**Figure 1a.**
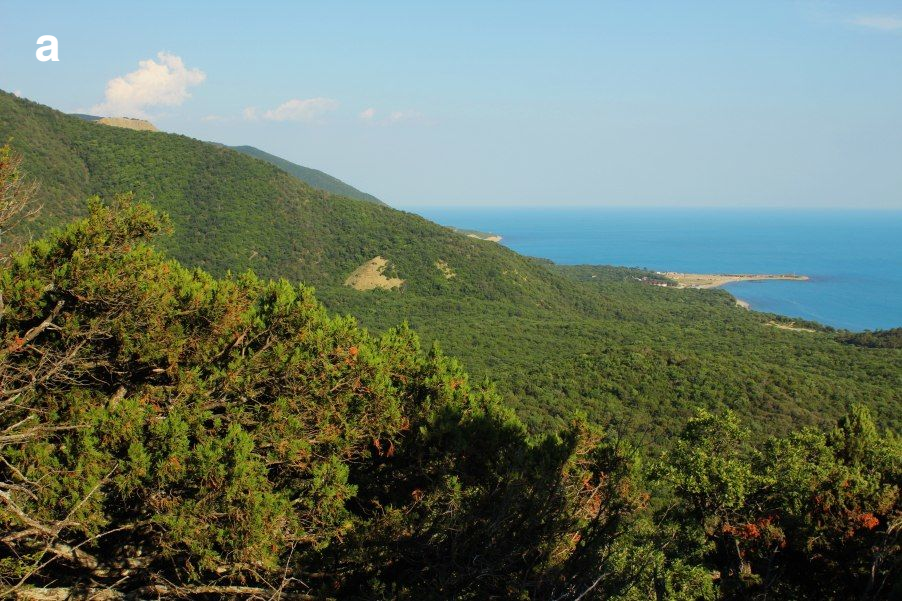
A view of the coast of Utrish Nature Reserve

**Figure 1b.**
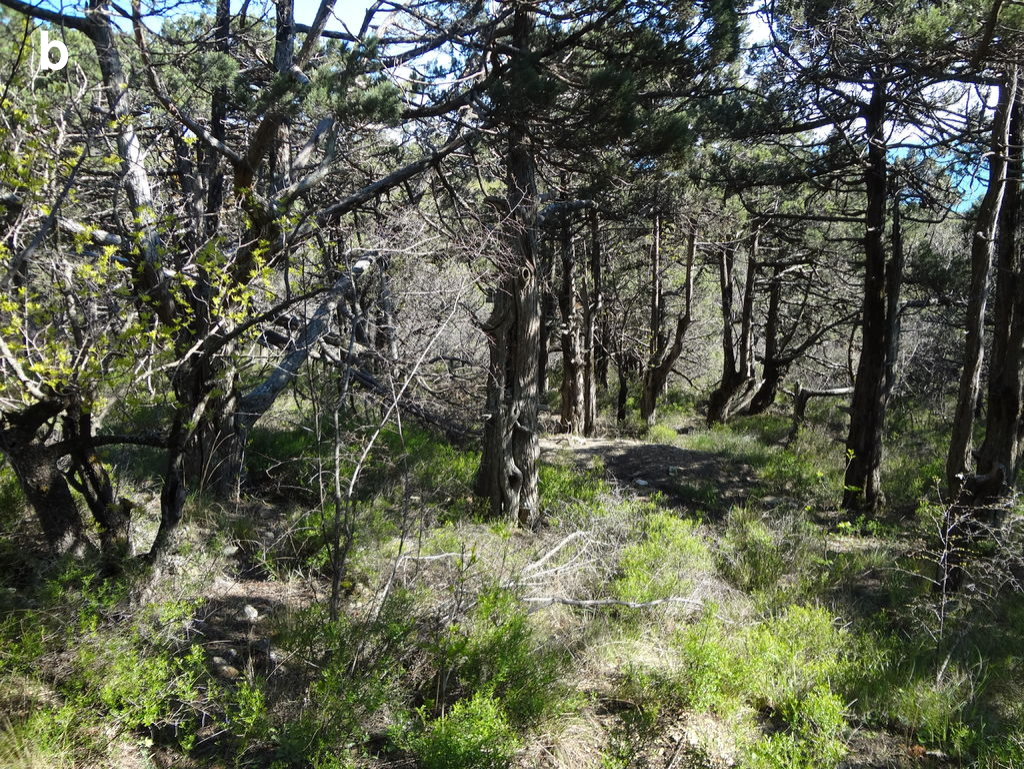
*P. mutica* - *Juniperus* shrubland on the dry slope

**Figure 1c.**
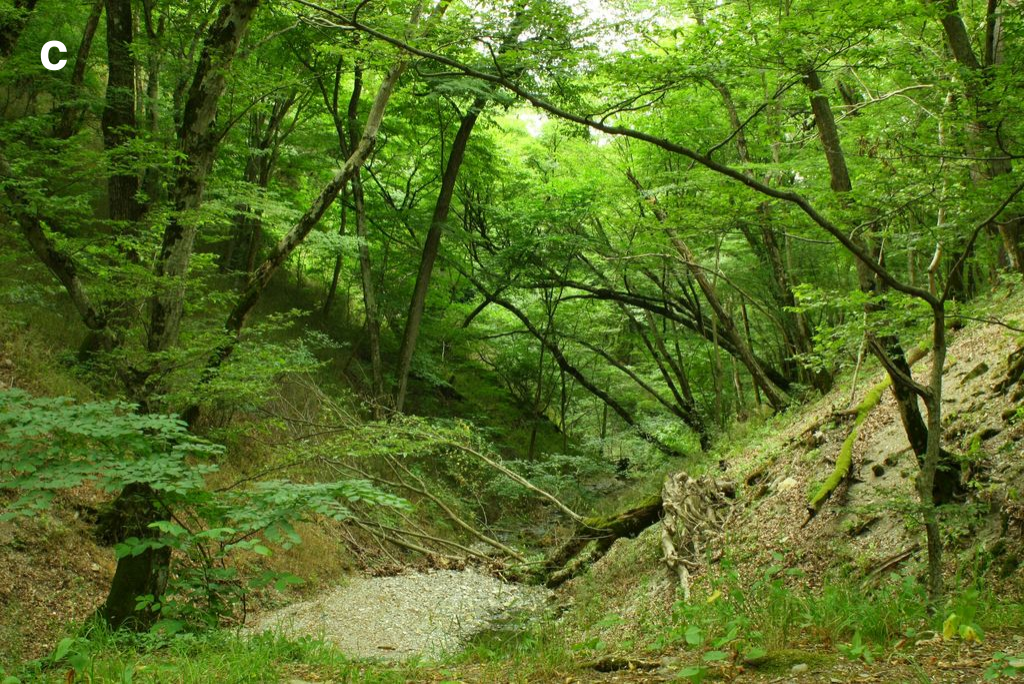
*Q. petraea, F. orientalis, C. caucasica* forest

**Figure 1d.**
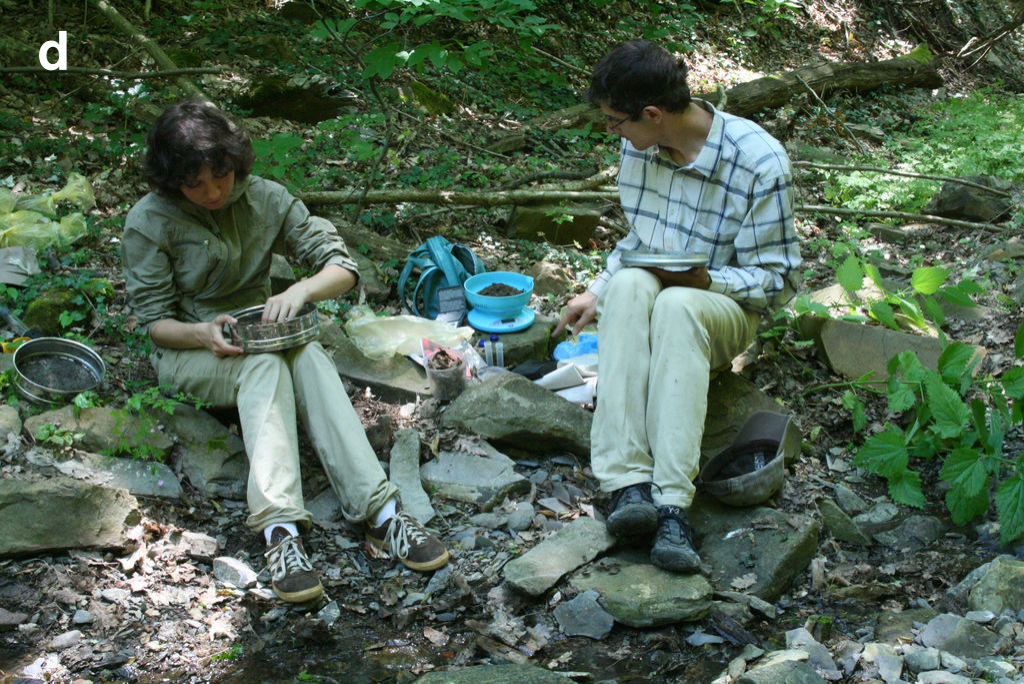
T.Yu. Lushnikova and K.B. Gongalsky collecting invertebrates near a stream in *F. orientalis - C. caucasica* forest

**Table 1.** The description of sampling plots at the Abrau Peninsula, N-W Caucasus.

**No.**	**Habitat**	**Coordinates and elevation (m a.s.l.)**
{**1**}	*Quercus petraea, Fagus orientalis, Carpinus caucasica* forest	44°45'15'' N, 37°29'53'' E; 295
{**2**}	*Q. petraea, F. orientalis, C. caucasica* forest	44°44'13'' N, 37°28'46'' E; 153
{**3**}	*Quercus pubescens* – *C. caucasica* forest	44°42'51'' N, 37°28'45'' E; 47
{**4**}	*Q. pubescens* – *C. caucasica* forest	44°42'50'' N, 37°27'01'' E; 8
{**5**}	Mixed *Q. pubescens*, *C. caucasica, Juniperus excelsa* forest with *Achnatherum bromoides*, *Physospermum cornubiense*, *Dictamnus albus*	44°42'38'' N, 37°27'31'' E; 10
{**6**}	*Q. pubescens* forest with *Carpinus orientalis* and steppe Graminaea species.	44°42'34'' N, 37°27'25'' E; 6
{**7**}	Steppe meadow with *Vitex agnus-castus*, *Q. pubescens* and *C. orientalis*	44°42'29'' N, 37°27'28'' E; 2
{**8**}	*Q. pubescens – C. caucasica* forest near the Lake Sukhoy Liman	44°45'14'' N, 37°27'26'' E; 308
{**9**}	Mixed *Q. pubescensC. caucasica, Fraxinus excelsior* forest	44°41'44'' N, 37°29'06'' E; 9
{**10**}	*Tilia begoniifolia* – *Q. petraea* forest with the oriental hornbeam and *Acer laetum*	44°43'31'' N, 37°29'04'' E; 85
{**11**}	*F. orientalis* forest	44°42'56'' N, 37°28'50'' E; 54
{**12**}	*Q. pubescens* – *C. orientalis* forest with *Cotinus coggygria*	44°42'48'' N, 37°28'39'' E; 50
{**13**}	*Q. pubescens* – *C. orientalis* forest with *Juniperus oxycedrus*, *J. excelsa*, *Ruscus ponticus* and Graminaea.	44°42'39'' N, 37°28'37'' E; 31
{**14**}	Marine station RAS; mixed *Q. pubescens, C. orientalis, Pistacia mutica* forest with *J. excelsa* and fruit-trees (*Prunus avium*, *P. armeniaca*, *Ficus carica*, *Morus* sp.).	44°42'21'' N, 37°28'15'' E; 16
{**15**}	*F. orientalis* forest with *C. caucasica*	44°45'02'' N, 37°30'05'' E; 273
{**16**}	*T. begoniifolia* forest with *F. orientalis* and *C. caucasica*	44°44'27'' N, 37°29'53'' E; 195
{**17**}	*F. orientalis* forest with *Q. petraea*	44°43'46'' N, 37°29'13'' E; 116
{**18**}	*Q. petraea* forest with *A. laetum*	44°44'02'' N, 37°29'32'' E; 172
{**19**}	*C. caucasica* forest with *Q. petraea*	44°44'10'' N, 37°28'47'' E; 149
